# Dietary composition and fasting regimens differentially impact the gut microbiome and short-chain fatty acid profile in a Pakistani cohort

**DOI:** 10.3389/fsysb.2025.1622753

**Published:** 2025-10-17

**Authors:** Farzana Gul, Hilde Herrema, Aqsa Ameer, Mark Davids, Arshan Nasir, Konstantinos Gerasimidis, Umer Zeeshan Ijaz, Sundus Javed

**Affiliations:** ^1^ Department of Biosciences, COMSATS University Islamabad, Islamabad, Pakistan; ^2^ Department of Experimental Vascular Medicine, Amsterdam University Medical Centers, Amsterdam, Netherlands; ^3^ School of Medicine, Dentistry and Nursing, Glasgow Royal Infirmary, Glasgow, United Kingdom; ^4^ Water and Environment Research Group, University of Glasgow, Mazumdar-Shaw Advanced Research Centre, Glasgow, United Kingdom; ^5^ Department of Molecular and Clinical Cancer Medicine, University of Liverpool, Liverpool, United Kingdom; ^6^ University of Galway, Galway, Ireland

**Keywords:** diet, gut microbiota, intermittent fasting, Ramadan fasting, SCFA

## Abstract

**Purpose:**

Fasting is known to have beneficial effects on human physiology and health due to changes in gut microbiota and its associated metabolites. We investigated the effects of intermittent and Ramadan fasting on the gut microbial composition, diversity, and short-chain fatty acid (SCFA) profile in a Pakistani population.

**Methods:**

Paired fecal samples—a total of 29 for Ramadan fasting (divided into three groups, before and after completion and after 3 months) and 22 for intermittent fasting (divided into two groups, day 1 and day 10)—were collected for both 16S rRNA microbiome profiling and SCFA analysis. Study volunteers also provided a detailed questionnaire about the dietary regimen before and during the fasting period. Descriptive statistics were applied to ascertain variations in the gut microbiome and SCFAs attributable to changes in food consumption during fasting.

**Results:**

Ramadan fasting increased the bacterial taxonomic and functional diversity and decreased the abundance of certain harmful microbes such as Blautia, *Haemophilus*, Desulfovibrio, Lachnoclostridium, and Porphyromonas. Intermittent fasting showed increased abundance of Prevotella, *Lactobacillus,* and Anaerostipes. Ramadan fasting also led to a significant increase in SCFAs including C7, iC4, and iC6, accounting for variability in microbial composition and phylogeny, respectively. In intermittent fasting, C5, iC5, and iC6 contributed to variability in microbial composition, phylogeny, and function, respectively.

**Conclusion:**

Both fasting regimens impacted gut microbiome and metabolic signatures, but Ramadan fasting showed a more drastic effect due to the 30 days compliance period and water restriction than intermittent fasting. Ramadan fasting also improved metabolic health by increasing the abundance of SCFA-producing microbes. With Ramadan fasting, most microbial taxa reverted to their prefasting state after resumption of normal feeding patterns with few exceptions, indicating impact on microbial niche creation with prolonged fasting regimens that benefit *Enterococcus, Turibacter*, and *Klebsiella* colonization. The dietary regimen adopted during fasting, especially the consumption of high-fat-content food items, accounted for persistent gut microbial changes.

## Introduction

Gut microbiota are an important component of the human body and are involved in metabolic functions such as the fermentation of complex polysaccharides into short-chain fatty acids and the synthesis of essential amino acids and vitamins (Khan et al., 2022; Li et al., 2020). Gut microbiota are influenced by various environmental, psychological, and genetic factors (Clarke et al., 2019; Gul et al., 2024). Among these, diet is considered an important factor that changes the gut’s microbial diversity and composition (Beam et al., 2021). A high caloric diet with a high fat and carbohydrate content increases the abundance of certain pathogenic bacterial species that alter the gut barrier function, enhance inflammation, and cause hormonal imbalance and dyslipidemia, leading to diabetes, cancer, and cardiovascular and inflammatory diseases (Rinninella et al., 2020). A review based on an Asian population also highlighted that a high carbohydrate or high-fat diet is associated with alterations in gut microbiota and their metabolites, which could lead to chronic heart failure (Kona’atul et al., 2025). On the other hand, a high-fiber diet upregulates butyrate-producing species and acetate-producing bacteria that can reduce gut dysbiosis (Alou et al., 2016; Marques et al., 2017). A calorie-restricted diet significantly reduces the *Firmicutes-*to-*Bacteroidetes* ratio and enriches beneficial microorganisms such as *Bacteroides*, *Roseburia*, *Faecalibacterium*, and *Clostridium XIVa* (Zhang et al., 2021).

Increasing evidence indicates that different dietary regimens with varied feeding patterns can also shift the gut microbiota, directly impacting host metabolic activity by regulating blood glucose, insulin signaling, and inflammation (Angoorani et al., 2021; Forslund, 2023). Intermittent fasting is gaining in popularity worldwide as a weight-reduction and metabolism-boosting strategy. In addition, intermittent fasting also improves blood pressure regulation and cardiovascular health, increases renal function, and lowers brain natriuretic peptide levels (Khan et al., 2022; Li et al., 2017). Different studies have shown that intermittent fasting also has advantageous effects on reshaping the gut microbiome (Ducarmon et al., 2023; Mesnage et al., 2019). For instance, increased microbial diversity, especially enrichment of *Bacteriodeacea* and *Prevotellaceae*, has been reported after time-restricted feeding (Popa et al., 2023; Zeb et al., 2020). However various intermittent fasting regimens are practiced, including time-restricted feeding, alternate day fasting, and calorie-restricted feeding, that may have a variable impact on the host gut’s microbiota and metabolism (Llewellyn-Waters and Abdullah, 2021).

Ramadan fasting is another popular fasting regimen in which millions of Muslims observe a fast from sunrise to sunset in the month known as “Ramadan” (Mousavi et al., 2022). This type of prolonged fasting is also known to exert beneficial effects on the gut microbiome, with improved health outcomes like the regulation of blood glucose and BMI (Ali et al., 2021; Jo et al., 2023; Maifeld et al., 2021; Ozkul et al., 2020; Su et al., 2021). Ramadan fasting is also known to have preventive effects against metabolic syndrome, cancers, inflammation, Alzheimer’s disease, and various neuropsychiatric disorders (Mindikoglu et al., 2020).

As majority of the studies lack a direct comparison between fasting regimens in the same ethnic group, with similar sociodemographic parameters, we designed our study to investigate the effects of both dietary regimens (10 days intermittent versus 30 days prolonged Ramadan fasting) on the gut microbiome of healthy Pakistani individuals. Furthermore, a majority of fasting studies report a shift in microbial communities of major SCFA producers (Ali et al., 2021; Guo et al., 2021; Maifeld et al., 2021). Therefore, fecal SCFA profiles were also assessed in parallel with the gut microbiome to observe metabolite signatures. As the highest impact was observed after prolonged fasting, samples were collected 3 months after the completion of fasting, in addition to pre- and post-fasting periods to observe whether the regimen had a long-term impact on the gut microbiome and metabolomic profiles.

## Methods

### Study participants’ identification, screening, and recruitment

For Ramadan fasting, ten volunteer participants (six female and four male) aged 18–40 (mean age 27.5 + 3.77), and for intermittent fasting, eleven participants (six female and five male) aged 18–40 (mean age 29.5 + 5.48), were identified for the study. All participants belonged to four major geographic regions (Punjab, KPK, AJK (Azad Jammu and Kashmir) and ICT (the Islamabad Capital Territory)) and were screened via questionnaire based on the following inclusion/exclusion criteria. The major exclusion criteria were age <18 or  >40, body mass index (BMI) <18 or >30 kg/m^2^, antibiotic or multivitamin intake within the last 3 months, any prior clinical history of chronic or acute infections or other diseases, pregnant or lactating women, or women with irregular menstrual cycles (i.e., <21 or >35 days apart). The study was approved by the Ethics Review Board (ERB No. CUI/Bio/ERB/12–09/22) at COMSATS University Islamabad. All study participants provided written informed consent to participate and a detailed extensive questionnaire about each participant’s medical history, dietary habits before, during, and after Ramadan and intermittent fasting, sleeping pattern, and routine lifestyle.

### Sample collection

For Ramadan fasting, participants observed fasting of 15 h from sunrise to sunset for 30 days from 13 April to 12 May 2021. Stool samples (one for microbiome analysis and one for SCFA analysis) were collected in the morning at three time points; 1) 1 week before the start of Ramadan, 2) after completion of Ramadan (30 days), and 3) 3 months after completion of Ramadan from every individual. All participants collectively provided 58 samples (29 for microbiome and 29 for SCFA analysis) instead of 60 because one participant skipped sample donation at time point 2. For intermittent fasting, participants were restricted to not taking any solid food for 12 h during the night for 10 days. Stool samples in the morning were collected at two time points: 1) before the start of intermittent fasting and 2) after completion of the intermittent fasting period of 10 days. All participants collectively provided 44 samples (22 for microbiome and 22 for SCFA analysis). For microbiome analysis, all participants were given uBiome Explorer kits and briefly instructed on stool sampling methodology. These kits follow the protocols outlined by the NIH Human Microbiome Project (Keitel et al., 2010), available online at http://www.fda.gov/cder/guidance/959fnl.pdf (accessed 22 August 2023). To minimize the risk of selective bacterial overgrowth, particularly *Gammaproteobacteria* (Amir et al., 2017) at room temperature, participants were provided with standardized instructions and guided to wash their hands prior to sample collection. Using sterile swabs or wipes, small amounts of fecal material were transferred into vials containing lysis and stabilization buffer, enabling the preservation of DNA at room temperature. These sample containing kits were then shipped to the Microbiota Centre of Amsterdam (MiCA) in the Netherlands for subsequent downstream processing. For SCFA analysis, all participants were provided sterile stool collection containers. First bowel movement stool samples in the morning were collected by each participant. All samples were stored at −20 °C at CUI prior to shipping to the University of Glasgow, Scotland, UK, for subsequent steps.

### DNA extraction and PCR amplification

DNA extraction and PCR amplification were performed in the Microbiota Centre of Amsterdam (MiCA). First, the sample collection tubes were centrifuged at 14,000 RPM/18,626 RCF (fixed angle) for 10 min at room temperature, and stabilizing buffer was removed. Next, DNA from fecal samples was extracted using a repeated bead beating protocol and purified using Maxwell RSC Whole Blood DNA kits (Costea et al., 2017). Purified DNA concentration was measured using the Qubit®dsDNA BR Assay with 96-well plate (Invitrogen—Carlsbad, California, United States). Four sample collection kits containing only solubilizing buffer with no stool samples were used as negative control and were followed for the same extraction steps. V3–V4 amplicon sequencing was selected based on its established utility as the most appropriate choice, with low error propagation in Illumina sequencers (Callahan et al., 2016; Caporaso et al., 2010; Kozich et al., 2013). The V3–V4 region of the 16S ribosomal RNA (rRNA) gene was amplified with a single-step PCR protocol using universal primers B341 F and B806R. Ampure XP beads were then used to purify the amplicon libraries, and purified products were pooled equimolarly (Mills et al., 2021). The library was sequenced with an Illumina MiSeq platform using v3 chemistry with 2 × 250 cycles.

### Bioinformatics

Abundance tables were generated using the DADA2 denoising algorithm (Callahan et al., 2016) in QIIME2 workflow (Caporaso et al., 2010) to recover amplicon sequencing variants (ASVs). The details of the bioinformatics steps are provided at https://github.com/umerijaz/tutorials/blob/master/qiime2_tutorial.md and are similar to the materials and methods we previously described (Mills et al., 2021). The samples for all three studies were pooled to generate a single abundance table (n = 176 samples × P = 4,751 ASVs). These samples included four negative controls, which were later used to identify and remove contaminants (11 ASVs) by employing the prevalence method in R’s “decontam” package (Davis et al., 2018). Additionally, we used the PICRUSt2 algorithm (Douglas et al., 2020) within the QIIME2 environment (using the standard parameters) to recover the metabolic potential of ASVs and generate abundance tables for both KEGG enzymes and MetaCyc pathways at sample level. The reference database in PICRUSt2 has known metabolic functions for ∼20,000 genomes, and only four ASVs out of 4,751 in this study did not match (a very high alignment of ∼99% ASVs), increasing our confidence in the recovered metabolic predictions. ASVs were classified using the SILVA SSU Ref NR database release v.138 (Quast et al., 2012), and then taxonomic information was combined with the abundance table to generate a BIOM file. The rooted phylogenetic tree, also generated using the QIIME2 framework, along with the above 38 BIOM file and the functional tables from PICRUSt2, were then used in the downstream statistical analyses in R.

### Statistics

As a preprocessing step, we removed typical contaminants such as *Mitochondria*, *Chloroplasts*, and any ASVs that were unassigned at all levels, as per recommendations given at https://docs.qiime2.org/2022.8/tutorials/filtering/, and we also filtered out samples that were not relevant to this study (or were < 2,000 reads), giving abundance tables of: n = 19 samples × P = 1,214 ASVs for the intermittent fasting study (sample-wise read statistics as [minimum: 16,294; 1st quartile: 19,858; median: 22,185; mean: 22,900; 3rd quartile: 24,846; maximum: 36,000]) and n = 26 samples × P = 1,458 ASVs for the Ramadan fasting study (sample-wise read statistics as [minimum: 7,766; 1^st^ quartile: 16,032; median: 19,742; mean: 19,565; 3rd quartile: 22,041; maximum: 38,171]). Note that 11 subjects provided two samples each for the intermittent fasting study, and 10 provided three samples each for the Ramadan fasting study, with the above numbers being the final number of samples making it to the statistical analysis. Further details are provided in the Supplementary Material.

### Short-chain fatty acid analysis

For short-chain fatty acid (SCFA) analysis, gas chromatography–mass spectrometry (GC–MS) (Agilent Technologies 7820A) was used. Stool samples were collected in containers and stored at −20 °C. The samples were then transferred to bijoux tubes containing 1 mL of 1M NaOH and shipped to the University of Glasgow for further processing. For freeze-drying, small holes were made in tube lids with sterile needles. All the tubes were kept at −80 °C for 4–5 h. The tubes were then kept in a freeze-dryer for 24 h. Next, frozen samples were crushed and transferred to new tubes. For SCFA extraction, samples were prepared by weighing out 100 mg of the freeze-dried sample in Corning tubes, and 300 μL of distilled water was added to each tube. Then, 100 µL of internal standard (2-ethyl butyric acid) was added in each sample to account for any losses. Next, 100 µL of orthophosphoric acid was added, and the tubes were vortexed to mix the freeze-dried sample with the added contents. We then added 1.5 mL of diethyl ether to each tube and vortexed them for 1 minute on an IKA shaker at a speed of 1500 x g. The mixture was separated into two layers: i) upper organic and ii) lower aqueous phases. The top organic layer was extracted into separate tubes, 1.5 mL of diethyl ether was added into the tubes, and they were vortexed. These steps were repeated twice, and the upper layer was extracted each time. The extracted samples were transferred into small vials and covered with crimp top for GC analysis. Each vial was labeled properly, and six external standards were also included for GC. Vials along with the standards were then loaded on the auto sampler tray on the GC. A vial with ether only was used as a blank during the run to ensure no residual carry-over between samples. After putting all samples, standards, and blank in location, GC was run. Statistical analysis details are provided in the Supplementary Material.

## Results

### Differences in microbial taxonomic and functional diversity in the Ramadan and intermittent fasting groups

**FIGURE 1:**
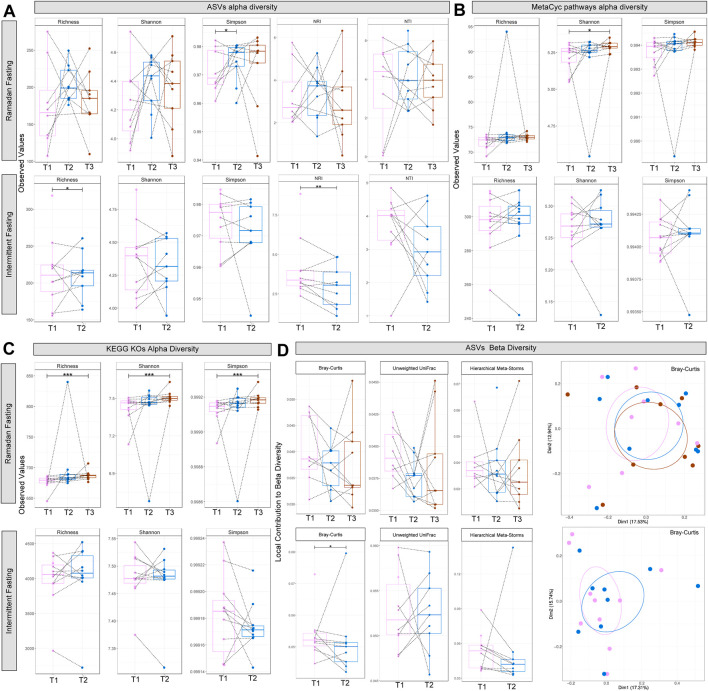
Diversity estimates. Alpha diversity comparison of bacterial ASVs **(A)**, recovered MetaCyc pathways **(B)**, and the KEGG KOs **(C)** from PICRUSt2 for both studies: *Ramadan* fasting study comprising three time points, and *intermittent* fasting study comprising two time points. Local contribution to beta diversity **(D)** figures using different distance measures represent average beta diversity as gray lines, while principal coordinate analysis (PCoA) plots with each axis shows the percentage variability explained by that axis, where ellipses represent 95% CI of standard error for a given time point. Lines connect different categories where values significantly differed (according to *one-way within ANOVA* accounting for subject IDs; *p < 0.05 **p < 0.01 ***p < 0.001).

The Ramadan fasting regimens impacted the gut microbial communities, and significant differences were observed in alpha diversity for Simpson at time point T2 (immediately after completion of 30 days fasting) in then Ramadan fasting group compared to T1 (before fasting). These differences in diversity remained consistent even after 3 months after completion of fasting (time point T3) (Figure 1A). Similarly, significant differences were observed in alpha diversity for bacterial richness at T2 (after 10 days fasting) in the intermittent fasting group. To understand the microbial ecological community assembly mechanisms, a null modeling approach was used (Figure 1A). The nearest taxon index (NTI) and nearest relative index (NRI) were used to determine whether the community structure was governed by strong environmental pressure (deterministic) or by competition among taxa (stochastic), respectively. In the Ramadan fasting group, no significant results were obtained, but the intermittent fasting group showed marginally significant results in terms of NRI. Note that an NRI/NTI value >0 suggests environmental filtering (i.e., microbiome assemblage driven by extraneous factors) with >2 values suggesting strong environmental pressures. In terms of functional alpha diversity, MetaCyc pathways and KEGG KOs showed significant changes at T2 and T3 in the Ramadan fasting group, but no differences were observed in the intermittent fasting group (Figures 1B,C). Next, by applying local contribution to beta diversity (LCBD) analysis, significant differences in the intermittent fasting group were observed for Bray–Curtis, while there were no significant differences in beta diversity for the Ramadan fasting group (Figure 1D).

### Signature microbiome in the Ramadan and intermittent fasting groups

**FIGURE 2:**
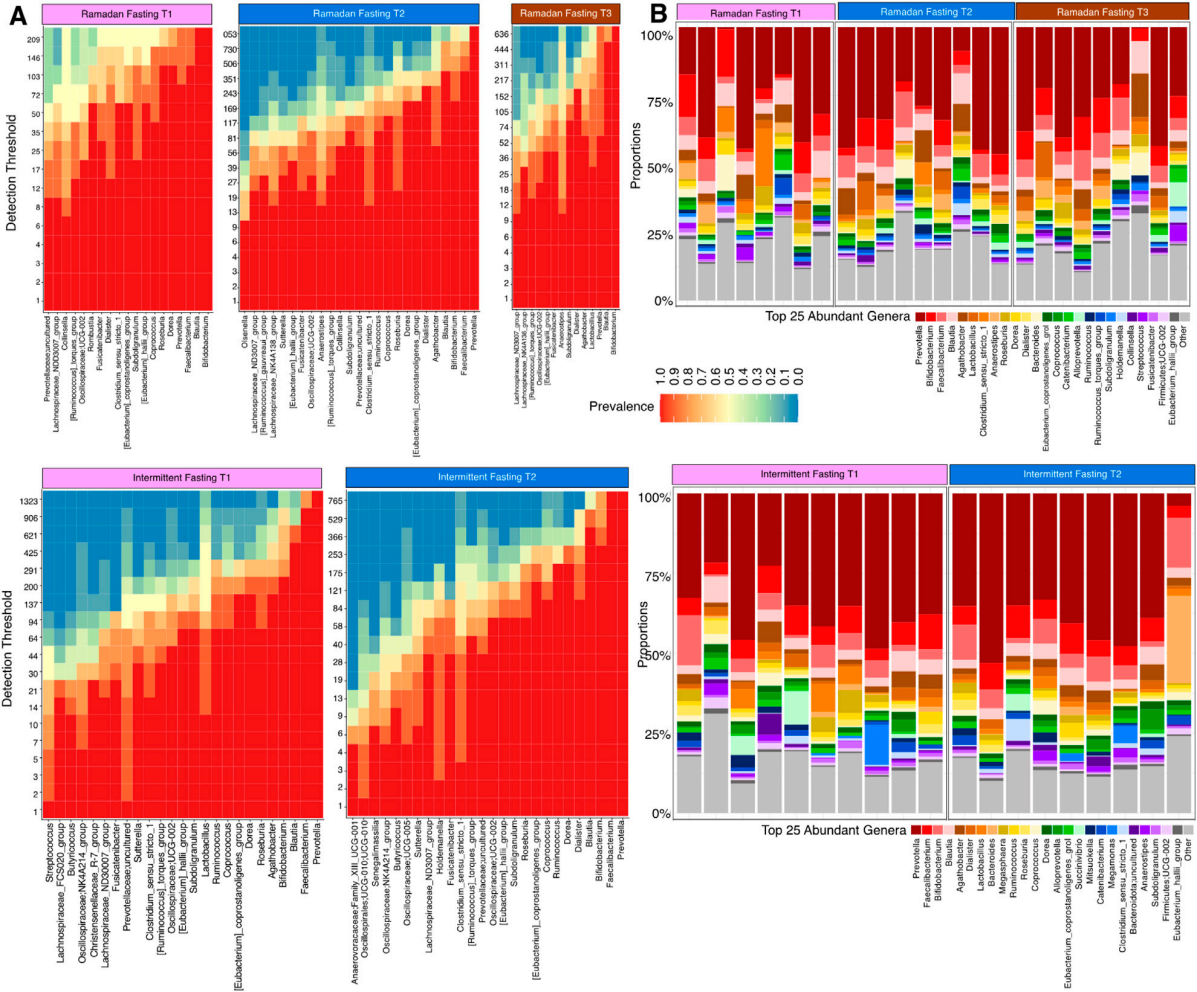
**(A)** Core microbiome at a minimum prevalence of 85% and at different abundance detection thresholds with values sorted to distinguish between low- (left side) and high- (right side) abundant core microbiomes in Ramadan fasting (top) and intermittent fasting (bottom) study. **(B)** Relative abundance of top-25 most abundant genera in both fasting groups.

The signature microbiome structure was identified using two approaches. The first was based on core microbiome analysis, which primarily considers taxa that have a minimum prevalence of 50% in samples at different time points in Ramadan and intermittent fasting groups (Figure 2A). Genera identified (Figure 2B) at different time points are highly abundant prevalent genera, while those on the left side of the figure are lowly abundant prevalent genera in both groups. It was observed that in the Ramadan fasting group, some genera were not present before fasting (T1) but appeared after 30 days of fasting (T2) —*Agathobacter*, *Anaerostipes, Sutterella, Lachnospiraceae_NK4A138_group, [Ruminococcus]_gauvreaui_group*, and *Olsenella*. Meanwhile, *Prevotella, Bifidobacter, Blautia*, and *Dialister* were present at both T1, T2, and T3. Only *Rombutsia* was observed to be present in T1, and it disappeared after fasting. On the other hand, certain genera seemed to be enriched post-fasting. These included *Lactobacillus* and *Lachnospiraceae_NK4A136_group*, which were prevalent in T3. In the intermittent fasting group, *Streptococcus, Lachnospiraceae_FCS020_group, Christensenellaceae_R-7_group, Lactobacillus*, and *Agathobacter* were abundant at T1, whereas *Dialister, Holdemanella, Oscillospiraceae; UCG-005, Senegalimassilia, Oscillospirales; UCG-010*, and *Anaerovoracaceae; Family_XIII_UCG-001* were observed only at T2 (Figure 2A).

An analysis of core genera in terms of their abundance in both fasting groups at different time points (Figure 2B) showed *Agathobater* and *Lactobacillus* abundance at T2, *Roseburia* at T2 and T3, and *Bacteroides* and *Anaerostipes* at T1 and T2 in individuals observing Ramadan fasting. In the intermittent fasting group, *Megasphaera, Dialister, Megamonas, Mitsuokella, Succinivibrio*, and *Roseburia* were present in abundance at T1. *Alloprevotella* and *Bacteroides* showed higher abundance at T2 (post-intermittent fasting).

### Differential microbial taxa in the Ramadan and intermittent fasting groups

Next, three differential analyses were performed to determine taxa with differential abundances between different time points of fasting in the Ramadan and intermittent fasting groups.

**FIGURE 3:**
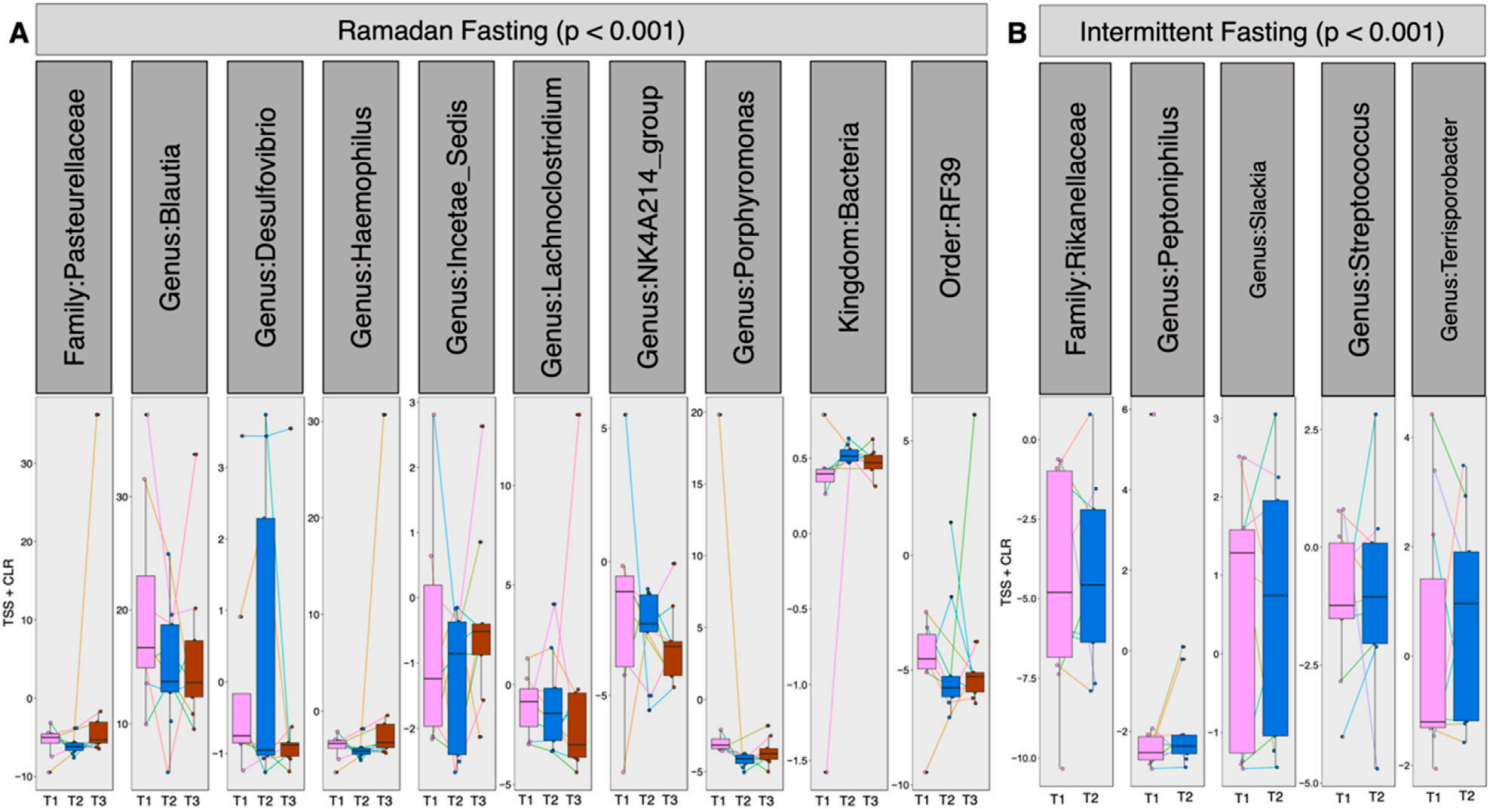
Differential abundance of taxa subset at pre- (T1), post- (T2), and 3 months after fasting (T3) for Ramadan **(A)** and intermittent **(B)** fasting groups using QCAT-C association test, which takes into account the paired nature of samples as originating from the same subject connected by lines. The values represent the TSS + CLR normalized abundances of individual taxa. The method returns a local p-value for individual taxa (all p < 0.05) as well as a global p-value shown in the panel strips.

First, a QCAT-C association test was used to determine the correlation between microbes due to the paired nature of the samples (Figure 3). This test visualizes the differentially abundant taxa at all taxonomic levels. Ramadan fasting showed that family *Pasteurellaceae* decreased after fasting (Figure 3A). The abundance of genera, including *Blautia, Haemophilus, Desulfovibrio, Lachnoclostridium*, and *Porphyromonas*, decreased at T2. Compared with Ramadan fasting, intermittent fasting showed an enrichment of family *Rikenellaceae* and some pathogenic genera such as *Peptoniphillus, Streptococcus*, and *Terrisporobacter* at T2. Only genus *Slackia* decreased after intermittent fasting (Figure 3B).

**FIGURE 4:**
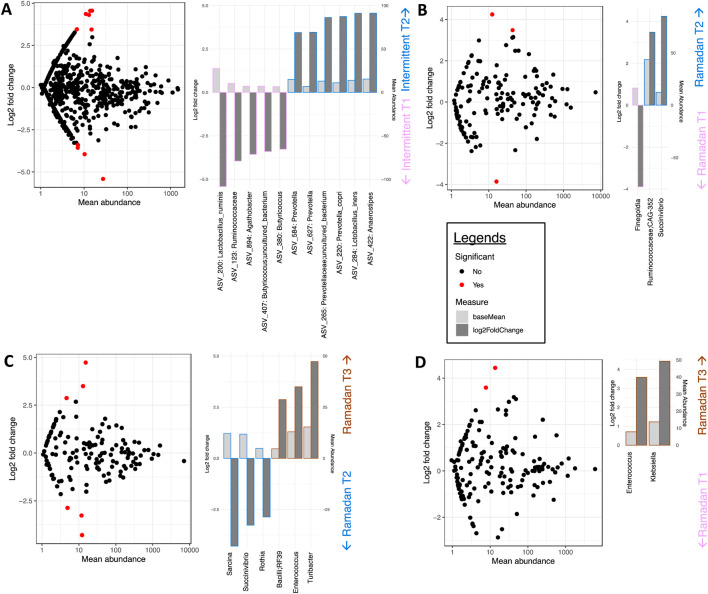
Taxa differential analysis on a temporal scale via DESeq2. **(A)** Intermittent T1 vs. T2, **(B)** Ramadan T1 vs. T2, **(C)** Ramadan T2 vs. T3, and **(D)** Ramadan T1 vs. T3. The left figure shows significant taxa in red that are at least two Log2 folds different, with the bar charts on the right side showing only significant Log2 fold changes in abundance between the groups (y-axis on the left and dark gray bar) and the mean abundance across all the samples (y-axis on the right and light gray bar). These are then sorted in terms of increasing Log2 fold change values. For the intermittent fasting study, we could find no significant variations at genus level, and therefore the algorithm was run at ASV level.

Second, DESeq was used to detect microbes that significantly varied between different time points (Figure 4). In intermittent fasting, *Lactobacillus_ruminis, Agathobacter*, and *Butyricoccus* were significantly abundant before fasting (T1) and showed decreased abundance after fasting (Figure 4A). Meanwhile, the abundance of *Prevotella_copri, Lactobacillus_iners* and *Anaerostipes* increased post-fasting (T2).

In Ramadan fasting, the gut microbiome prior to fasting (T1), immediately after the completion of 30 days fasting (T2), and 3 months after completion of fasting (T3) time points were compared to identify the differential microbes. Between T1 and T2, it was observed that *Finegoldia* was abundant at T1, while *Ruminococcaceae; CAG-352* and *Succinivibrio* showed increased abundance at T2 (Figure 4B). Meanwhile, *Sarcina* and *Rothia* decreased at T2, while *Enterococcus* and *Turibacter* were increased at T3 (Figure 4C). Similarly, *Enterococcus* and *Klebsiella* showed significant abundance at T3 compared to T1 (Figure 4D).

### Minimal subset ASVs contributing to beta diversity in the Ramadan and intermittent fasting groups

**FIGURE 5:**
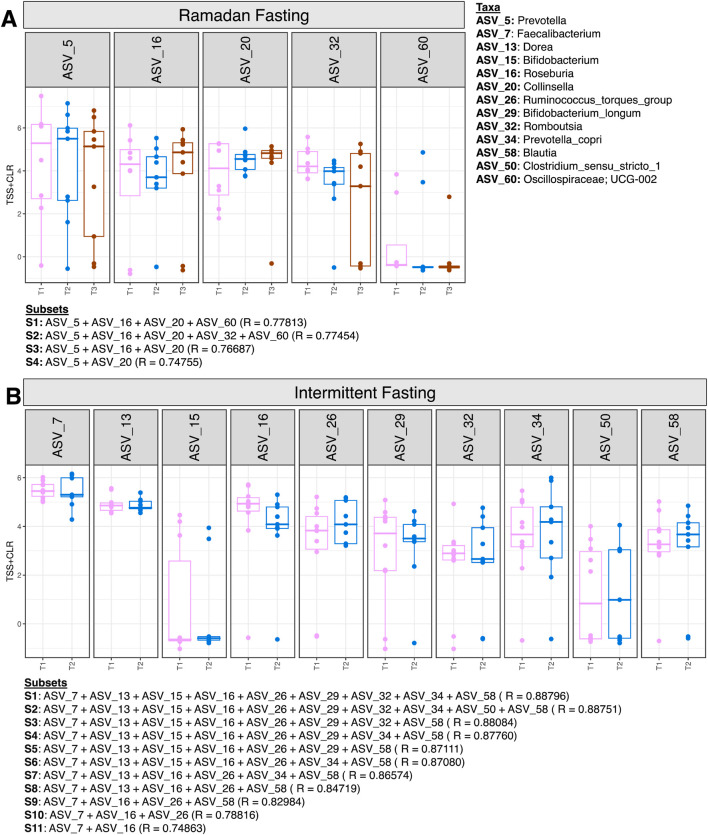
Expression of significant ASVs identified from the BVSTEP routine with samples of Ramadan fasting group **(A)** and Intermittent fasting group **(B)**. The information of ASVs from the top subsets is given below the figures with the correlation between Bray -Curtis distances between a particular subset, and the taxonomy of ASVs is given on the right side of the figure.

Third, the “BVSTEP” routine, a subset analysis, was performed to determine the minimal subset of ASVs which were causing major changes in beta diversity of the fasting samples at different time points (Figure 5). In the Ramadan fasting group, four ASVs were associated with major variations in beta diversity. *Prevotella* (ASV_5) and *Collinsella* (ASV_20) abundance increased at T2 (Figure 5A). *Prevotella* declined at T3, but *Collinsella* were in abundance even after 3 months of fasting. Meanwhile, *Roseburia* (ASV_16) showed reversion to baseline levels after 3 months of fasting after a decline in abundance immediately after fasting (T2). *Rombustia* (ASV_32) on the other hand decreased at T2 and remained consistently low in abundance at T3.

In the intermittent fasting group, *Faecalibacterium* (ASV_7), *Dorea* (ASV_13), *Roseburia* (ASV_16), *Bifidoobacterium_longum* (ASV_29), and *Rombustia* (ASV_32) decreased at T2 (Figure 5B).

### Changes in short-chain fatty acids associated with changes in microbial composition and function

Redundancy analysis was performed to identify the most important SCFAs associated with variation in beta diversity (Bray–Curtis distance, unweighted UniFrac, weighted UniFrac, and hierarchical meta-storms) of fasting samples (Supplementary Table S1). For Ramadan fasting, C7 (heptanoic acid) and iC4 (isobutyric acid) explained 5% and 6.4% variability in microbial composition, respectively (Table 1). iC6 (isopropyl hexanoic acid) accounted for 9.3% and 6% variability in terms of microbial composition and phylogeny, respectively. However, iC4 (isobutyric acid) showed significant variability (7%) in terms of microbial functions. In intermittent fasting, C5 (valeric acid), iC5 (isovaleric acid), and iC6 (isopropyl hexanoic acid) contributed to 10%, 9%, and 12% variability in terms of microbial composition, phylogeny, and function, respectively (Table 2).

**TABLE 1:** Redundancy analysis with both (forward and reverse) selection was performed to select the most important SCFAs that were associated with beta diversity changes in Ramadan fasting.

	Covariates	df	SS	R^2^	F	p
Bray–Curtis distance						
	**iC6**	**1**	**0.5587**	**0.09372**	**2.5097**	0.001 ***
	C7	1	0.3454	0.05793	1.5514	0.057
	**iC4**	**1**	**0.3823**	**0.06412**	**1.7171**	0.018 *
	Residual	21	4.6752	0.78422		
	Total	24	5.9615	1.00000		
Unweighted Uni-Frac						
	**iC6**	**1**	**0.4993**	**0.06566**	**1.6551**	0.003 **
	C6	1	0.3834	0.05042	1.2707	0.093
	iC4	1	0.3861	0.05077	1.2796	0.076
	Residual	21	6.3356	0.83315		
	Total	24	7.6044	1.00000		
Weighted Uni-Frac						
	iC4	1	0.0016092	0.09107	2.4032	0.061
	C2	1	0.0013292	0.07523	1.9851	0.099
	Residual	22	0.0147317	0.83370		
	Total	24	0.0176702	1.00000		
Hierarchical Meta-Storms						
	C2	1	0.002847	0.06621	1.7032	0.086
	**iC4**	**1**	**0.003376**	**0.07850**	**2.0193**	0.045 *
	Residual	22	0.036779	0.85528		
	Total	24	0.043003	1.00000		

Significance codes: 0 “***” 0.001 “**” 0.01 “*” 0.05 “.” 0.1 “ ” 1.

Initial set of variables considered are as follows (with those selected in the final PERMANOVA model and being significant shown in bold case): condition (T1/T2/T3), C2, C3, C4, **IC4**, C5, IC5, C6, **IC6**, C7, and C8. Here df, SS, and F are “degree of freedom,” “sum of squared errors,” and “F statistic,” respectively. Note that R^2^, if significant, is the percentage variability in composition and function explained (dependent on the choice of distance metric), e.g., for Bray–Curtis Distance and iC6, R^2^ value of 0.09372 implies 9.3% variability in composition explained by variation in iC6.

**TABLE 2:** Redundancy analysis with both (forward and reverse) selection to select the most important SCFAs that explain variation in community matrices in intermittent fasting.

	Covariates	df	SS	R^2^	F	p
Bray–Curtis distance						
	**C5**	**1**	**0.3346**	**0.10565**	**1.772**	0.033 *
	Residual	15	2.8325	0.89435		
	Total	16	3.1671	1.00000		
Unweighted Uni-Frac						
	**iC5**	**1**	**0.4130**	**0.09577**	**1.5888**	0.021 *
	Residual	15	3.8992	0.90423		
	Total	16	4.3122	1.00000		
Weighted UniFrac						
	iC6	1	0.0023075	0.11199	1.8918	0.113
	Residual	15	0.0182962	0.88801		
	Total	16	0.0206037	1.00000		
Hierarchical Meta-Storms						
	**iC6**	**1**	**0.0036575**	**0.12034**	**2.0521**	0.037 *
	Residual	15	0.0267350	0.87966		
	Total	16	0.0303925	1.00000		

Significance codes: 0 “***” 0.001 “**” 0.01 “*” 0.05 “.” 0.1 “ ” 1.

Initial set of variables considered are as follows (with those selected in the final PERMANOVA model and being significant shown in bold case): condition (T1/T2), C2, C3, C4, IC4, **C5**, **IC5**, C6, **IC6**, C7, and C8. Here df, SS, and F are “degree of freedom,” “sum of squared errors,” and “F statistic,” respectively.

### Fasting induced changes in gut microbiota and short-chain fatty acids

**FIGURE 6:**
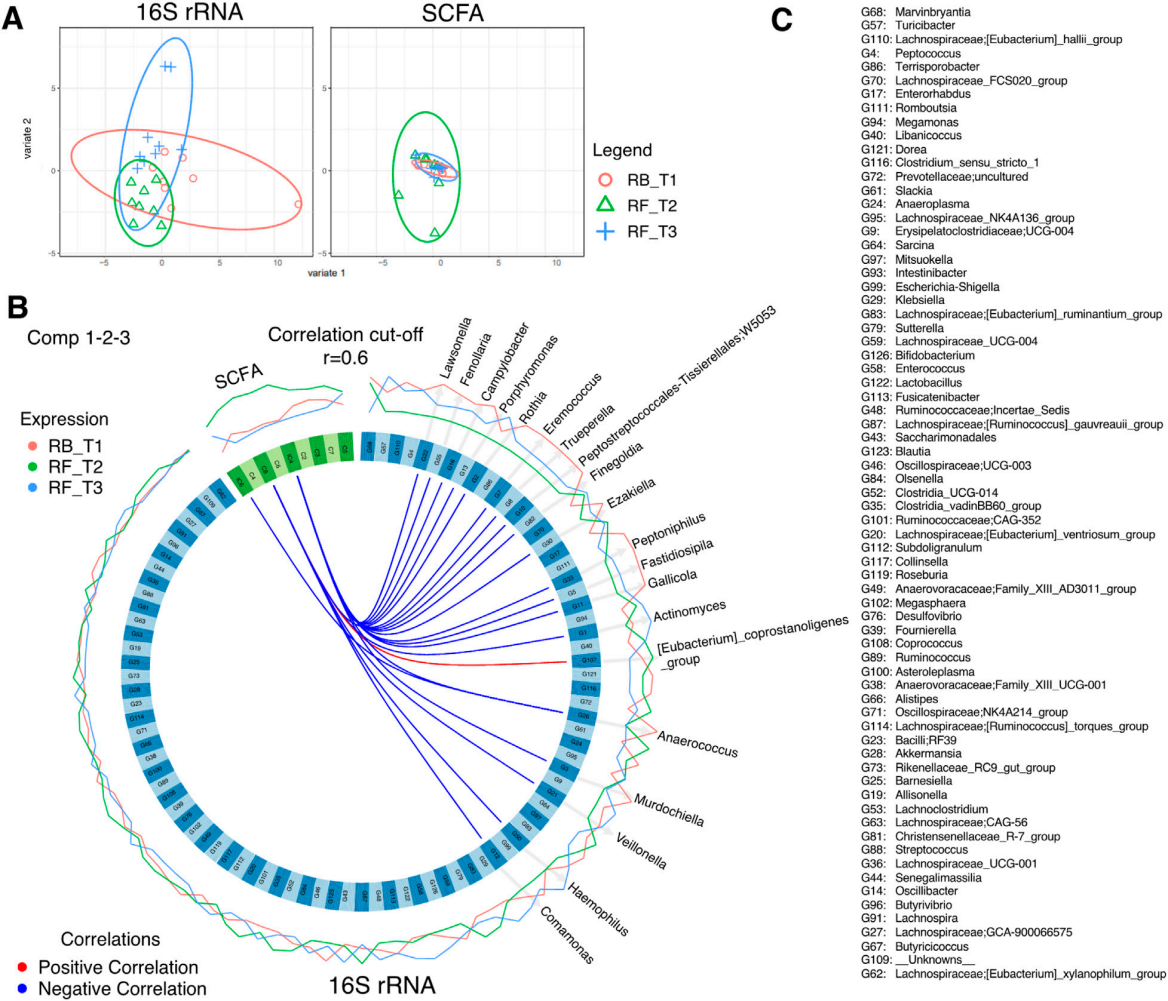
DIABLO analysis comparing changes in stool microbiota and SCFAs of Ramadan fasting samples. **(A)** The algorithm found three components, and this shows the reduced order representation of samples with ellipses representing 95% confidence interval and percentage variations explained by these components in axis labels for both microbiomes (Block: r16SrRNA) and SCFAs (Block: SCFA). **(B)** Significant correlations (|r|>0.6), whether positive (red) or negative (blue), between microbiome and SCFAs returned from the algorithm as a Circos plot. The expression levels of both r16SrRNAs and SCFAs for different categories (RB_T1, RF_T2, and RF_T3) are drawn around the circos plot as line plots. **(C)** The name of all discriminatory taxa are shown in **(B)**.

**FIGURE 7:**
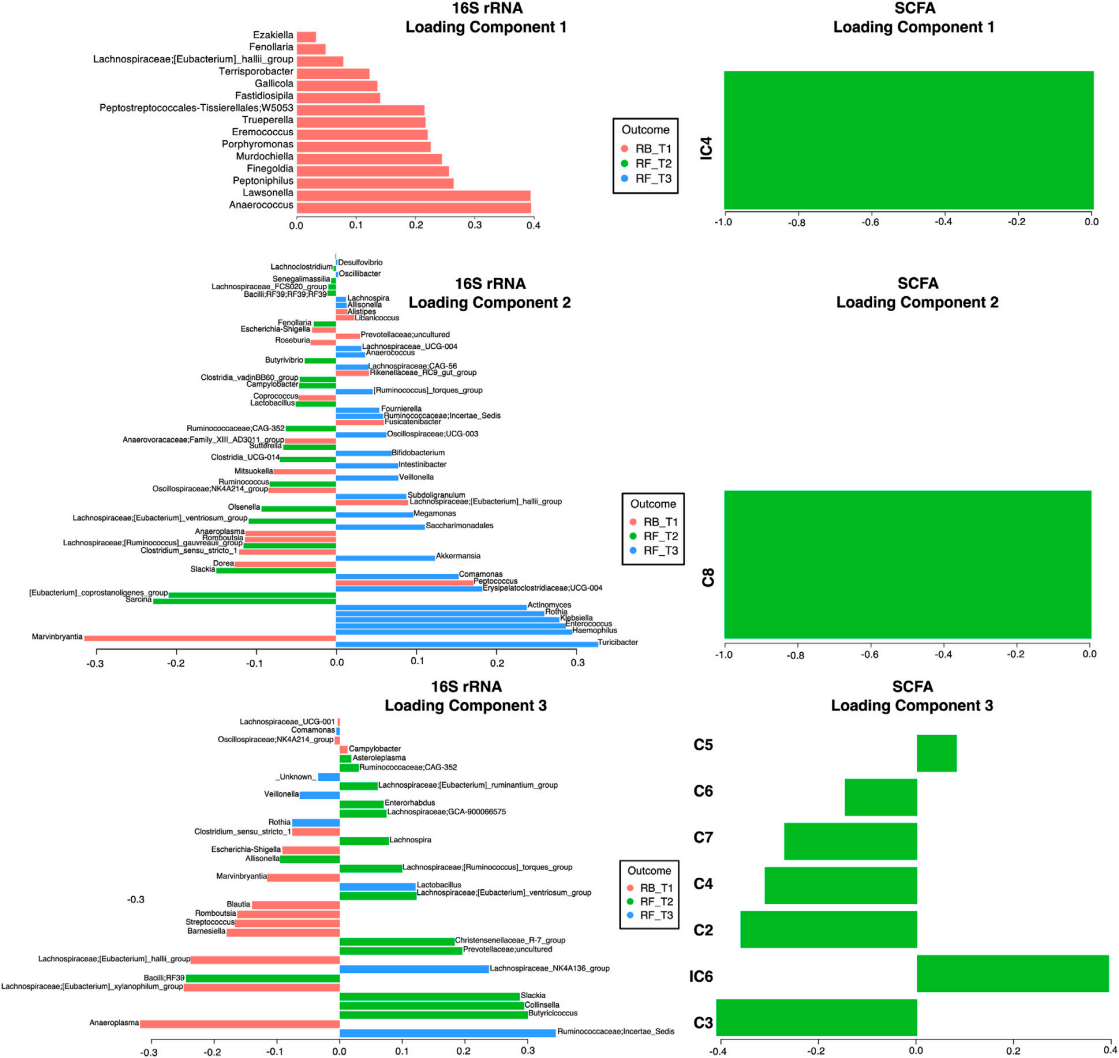
Non-zero components of loading vectors (for both microbiome and SCFA data) for Ramadan fasting samples. Color represents the categories (RB_T1, RF_T2, and RF_T3) in which the features have maximum expression/abundance.

**FIGURE 8:**
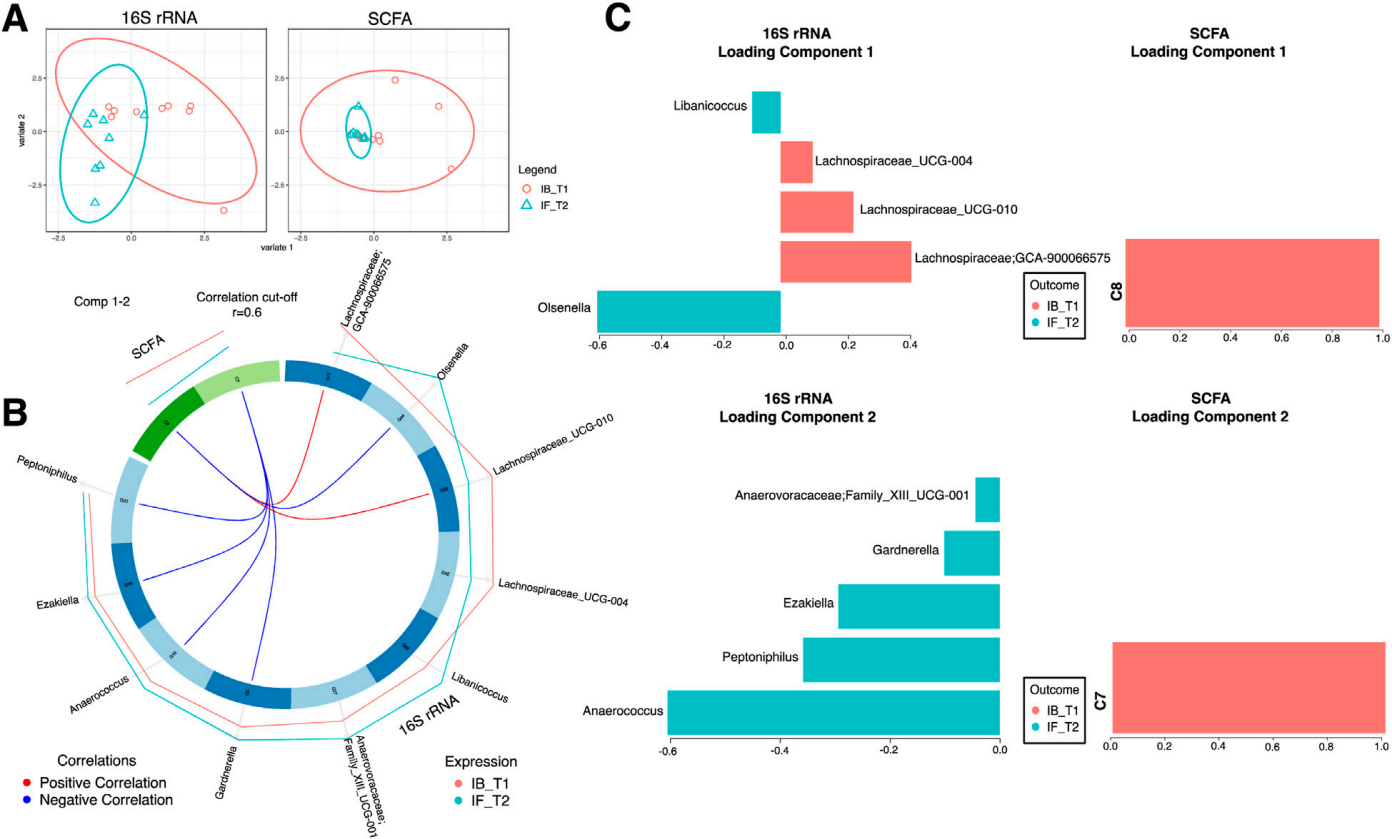
DIABLO analysis comparing changes in stool microbiota and SCFAs of intermittent fasting samples. **(A)** The algorithm found two components and shows the reduced order representation of samples with ellipses representing 95% confidence interval and percentage variations explained by these components in axis labels for both microbiomes (Block: r16SrRNA) and SCFAs (Block: SCFA). **(B)** Significant correlations (|r|>0.6), whether positive (red) or negative (blue), between microbiome and SCFAs returned from the algorithm as a Circos plot. The expression levels of both r16SrRNAs and SCFAs for different categories (IB_T1, and IF_T2) are drawn around the Circos plot as line plots. **(C)** Non-zero components of loading vectors (for both microbiome and SCFA data) with the color representing the categories (IB_T1, and IF_T2) in which the features have maximum expression/abundance.

To discriminate between changes in microbial communities and the metabolome at different time points of Ramadan and intermittent fasting, 16SrRNA and SCFA datasets were used. These were integrated with the DIABLO algorithm, which is a multi-group derivative of sparse PLS-DA. This algorithm reduces multiple features (bacteria and SCFA) to components (derived by a linear combination of features) that can be used to explain the variability within samples (Figures 6–8). The first step in identifying any associations between the changes in the microbiome and SCFA was to identify a reduced set of discriminant features that are maximally correlated across the datasets (16SrRNA and SCFA tables). The samples are then displayed based on these discriminant features as beta diversity diagrams for all three time points of Ramadan fasting (Figure 6A) and two time points of intermittent fasting (Figure 8A). From these discriminating features, those that are highly correlated across the datasets (|r|>0.6) are shown in Figure 6B for Ramadan fasting and Figure 8B for intermittent fasting. Figures 7 and 8C then show the coefficients of the loading components for the discriminating features (microbiome, left; SCFA, right) where the length of the bars represent importance whilst their color represent time point at which they have maximal abundance.

In the Ramadan fasting group, C2 (acetic acid), C3 (propionic acid), C4 (butyric acid), iC4 (isobutyric acid), C5 (valeric acid), C6 (caproic acid), iC6 (isocaproic acid), C7 (heptanoic acid), and C8 (octanoic acid) were increased immediately after completion of fasting (T2) (Figure 7). In the intermittent fasting group, only C8 and C7 showed decreased levels at T2 (Figure 8).

For, Ramadan fasting *Anaerococcus, Lawsonella, Peptoniphilus*, and *Finegoldia* were decreased after fasting, whereas *Sarcina*, *[Eubacterium]_coprostanoligenes_group, Olsenella, Ruminococcus* and *Butyricoccus*, *Collinsella*, and *Slackia* were increased (Figure 7). A positive correlation between octanoic acid and *[Eubacterium]_coprostanoligenes_group* at T2 was observed (Figure 6B), whereas octanoic acid was negatively correlated with *Veillonella* and *Haemophilus*. Isobutyric acid showed a negative correlation with *Peptoniphilus*, *Fastidiosipila*, and *Anaerococcus*, and isocaproic acid was negatively correlated only with *Anaerococcus*.

In the intermittent fasting group, *Libanicoccus, Olsenella, Ezakiella, Peptoniphilus*, and *Anaerococcus* abundance increased at T2 (Figure 8). A positive correlation between octanoic acid and *Lachnospiraceae; GCA-900066575* and *Lachnospiraceae_UCG-010* at T1 was observed (Figure 8B), whereas heptanoic acid was negatively correlated with *Peptoniphilus, Ezakiella*, and *Anaerococcus* at T2.

### Typical food items consumed during Ramadan and their association with changes in microbial composition, phylogeny, and function

**FIGURE 9:**
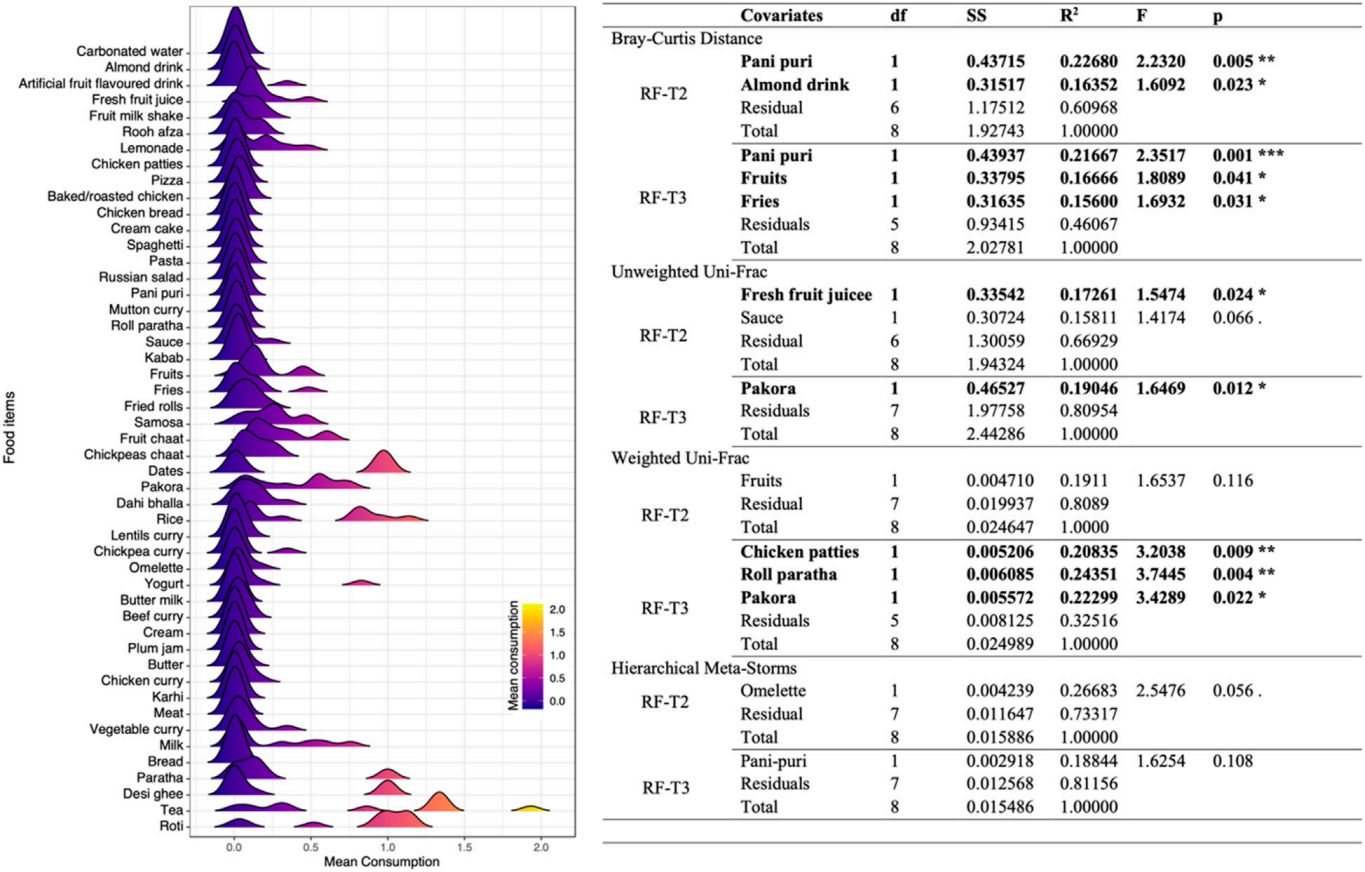
Association of mean consumption of food items with microbiome structure during Ramadan. Left panel shows the density profiles of mean consumption of food items of all participants. Right panel shows the redundancy analysis with both (forward and reverse) selections for the most important food items that explain variation in the community matrices in Ramadan fasting. The initial set of variables considered are the same as shown in the left panel (with those selected in the final PERMANOVA model and of significance shown in bold case). Here df, SS, and F are “degree of freedom”, “sum of squared errors”, and “F statistic”, respectively.

Based on the density plot for the mean consumption of food items by participants (Figure 9), many food items had a unimodal response, suggesting no variation in food intake among participants. The only difference in the dietary habits of participants was for food items that had multimodal density profiles, such as tea, roti, rice, desi ghee, yogurt, pakora (chick pea flour fritters), and milk. Redundancy analyses were then performed using samples at RF-T2 (immediately after Ramadan) and RF-T3 (3 months after Ramadan) to detect association with food items consumed. The reason behind choosing these two analyses was to see whether the consumption had short- or long-term effects and whether there are patterns that persist between the two cohorts. The redundancy analyses showed that pani puri and almond drink explained 22% and 16% variability in microbial composition, respectively, at RF-T2, while pani puri, fruit, and fries explained 21%, 16% and 15% variability, respectively, in microbial composition at RF-T3. Fresh fruit juice and pakora accounted for 17% and 19% variability in phylogenetic structure at RF-T2 and RF-T3, respectively. High-fat-content food items including chicken patties and roll paratha explained 20% and 24% variability when considering both composition and phylogeny using weighted UniFrac at RF-T3 only. No significant variations in microbial functions were recovered for food items at both time points.

### Correlation between short-chain fatty acids and mean consumption of food items at RF-T2 (immediately after Ramadan)

**FIGURE 10:**
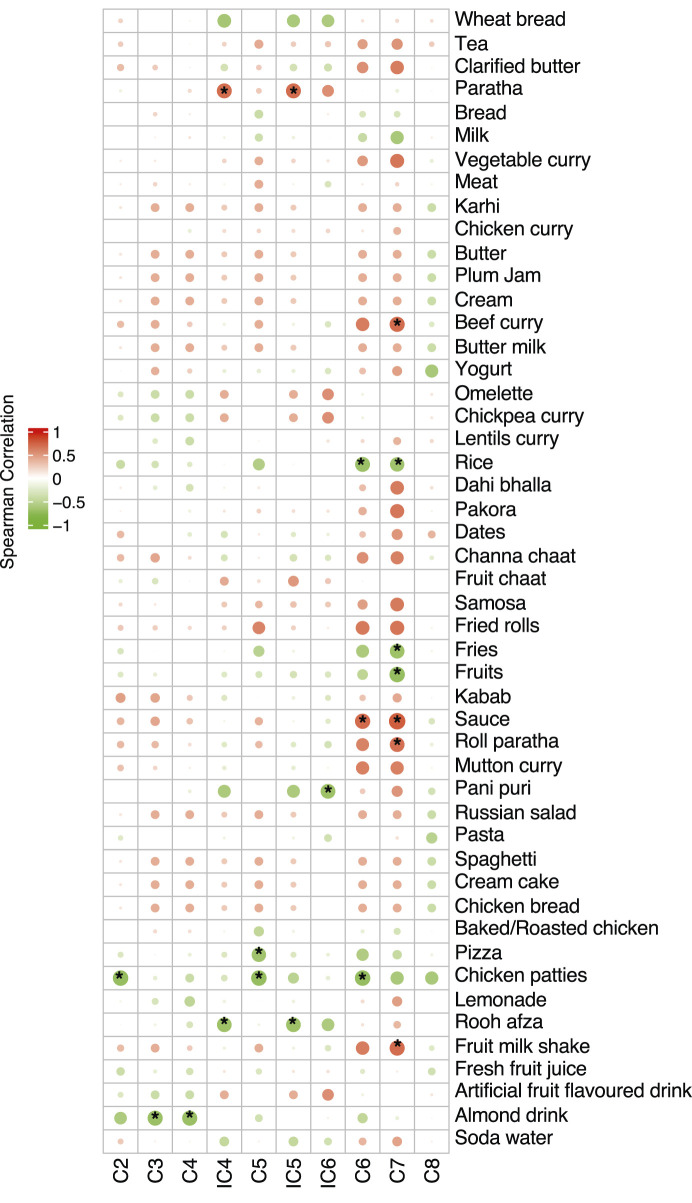
Correlation of mean consumption of food items with SCFAs during Ramadan. The size of the bubble shows the strength of the correlation and, if significant (p ≤ 0.05), is annotated with an asterisk (*). Positive correlations shown in red whilst negative correlations shown in green.

Correlation results (Figure 10) showed a significantly positive correlation between C7 and fruit milk shake, sauce, roll paratha and beef curry and a negative correlation with fruit, fries, and rice. C2, C5, and C6 were observed to be negatively correlated with chicken patties, and C3 and C4 were negatively correlated with almond drink. iC4 and iC5 showed a positive correlation with paratha and a negative correlation with rooh afza.

## Discussion

It is now well accepted that fasting not only impacts human physiology but it also influences gut microbial diversity and composition (Ali et al., 2021; Khan et al., 2022). In our study, both fasting regimens (Ramadan and intermittent fasting) caused changes in alpha diversity, coinciding with previously reported results based on time-restricted feeding (intermittent fasting) and Ramadan fasting (Jo et al., 2023; Pieczyńska-Zając et al., 2023). Ali and Abizari (2018) observed that Ramadan fasting is often accompanied not only by altered feeding patterns but also by nutritional composition. This dietary diversity is influenced by cultural and traditional norms and may therefore contribute to variations in gut microbial diversity observed in different studies. In terms of beta diversity, some animal and human studies have reported that intermittent fasting can cause changes in beta diversity (Li et al., 2020; Mousavi et al., 2022; Zeb et al., 2020). Meanwhile in Ramadan fasting, studies have reported different results that either cause changes in beta diversity (Ozkul et al., 2020; Su et al., 2021) or no significant differences (Ali et al., 2021; Ozkul et al., 2020; Su et al., 2021). These variations in results could be explained by differences in ethnicity as these studies are based on different populations—Pakistani and Chinese (Ali et al., 2021), Turkish (Ozkul et al., 2020), and just Chinese (Su et al., 2021)—and their dietary habits. In this study, we observed differences in the dietary habits of participants during Ramadan fasting for food items that had multimodal density profiles, including tea, roti, rice, desi ghee, yogurt, pakora, and milk. Increased consumption of these food items is often observed during Ramadan. Next, most prevalent and abundant microbial genera were studied in Ramadan and intermittent fasting groups. Ramadan fasting increased the prevalence of *Agathobacter*, *Anaerostipes, Sutterella, Lachnospiraceae_NK4A138_group, [Ruminococcus]_gauvreaui_group*, and *Olsenella*, which have previously been reported in post-Ramadan groups (Ali et al., 2021; Jo et al., 2023). *Ruminococcaceae* and *Lachnospiraceae* families mostly include butyrate-producing members. Apart from these, *Anaerostipes* is also considered an important taxon of the butyrogenic group (Bui et al., 2021). *Agathobacter* is also well known for its role in SCFA production, whereas *Sutterella* has been identified as having a beneficial role in human health as a blood sugar regulator (Oh et al., 2021; Wang et al., 2020). The prolonged effects of Ramadan fasting showed an enrichment of *Lactobacillus* and *Lachnospiraceae_NK4A136_group* which were prevalent even after 3 months of fasting. *Lactobacillus* are probiotic bacteria which have anti-inflammatory property and have been found to play an important role in immune regulation (Ding et al., 2017). *Lachnospiraceae_NK4A136_group* belongs to family *Lachnospiraceae*, and their potential beneficial role in gut health includes the production of SCFAs such as acetate and butyrate (Wu et al., 2020). *Roseburia*, *Bacteroides*, and *Anaerostipe* were also detected as the most abundant genera after Ramadan fasting. *Roseburia* has anti-inflammatory properties and is considered a marker of a healthy gut microbiome. Being a member of phylum *Firmicutes*, they produce butyrate, which helps maintain intestinal mucosa. An increased abundance of *Roseburia* after fasting is well reported in different studies (Ali et al., 2021; Ozkul et al., 2020; Pieczyńska-Zając et al., 2023). *Bacteroides* is another significantly enriched genus after fasting. They have the unique property of metabolizing complex polysaccharides and reducing the oxygen level in the gut (Ozkul et al., 2020).

Intermittent fasting increased the prevalence and abundance of gut-health-promoting microbes. For example, *Dialister* plays an important role in glucose metabolism and was previously detected as the most abundant genus in time-restricted feeding regimens (Barengolts et al., 2018; Zeb et al., 2020). *Holdemanella* promotes anti-inflammatory activity and improves glucose tolerance (Romaní-Pérez et al., 2021). *Senegalimassilia* also has anti-inflammatory properties and plays a vital role in protecting the intestinal barrier (Vázquez-Cuesta et al., 2023). *Megasphaera* belongs to the phylum *Firmicutes* and is known to produce SCFAs and control glucose levels in diabetic patients (De La Cuesta-Zuluaga et al., 2017; Shetty et al., 2013). *Succinivibrio* has fiber-degrading properties and has been reported to decrease after water-only fasting (only water intake and no food consumption) (He et al., 2019; Milani et al., 2016). Previously, less abundance of *Alloprevotella* has been observed after a time-restricted feeding pattern (Zeb et al., 2020).

Ramadan fasting decreased the abundance of potentially harmful *Pasteurellaceae*, *Blautia*, *Lachnoclostridium, Porphyromonas*, and *Desulfovibrio. Blautia* is associated with diabetes, obesity, the inhibition of insulin signaling, and inflammatory diseases (Liu et al., 2021). *Lachnoclostridium* produces harmful lipid compounds and decreases the levels of acetate, which could cause cardiometabolic diseases (Nogal et al., 2021). Decreases in *Blautia* and *Lachnoclostridium* during Ramadan fasting suggest a protective role of the fasting regimen against obesity and cardiometabolic diseases. These findings coincide with Saglam et al. (2023) on the effects of Ramadan fasting on the gut microbiome. Fasting has also been shown to regulate blood pressure and reduce the need for antihypertensive medications through changes in the gut microbiome (Maifeld et al., 2021). *Porphyromonas* is also a gram-negative pathogenic bacterium which causes periodontitis and inflammatory diseases (Zheng et al., 2022). *Desulfovibrio* are sulfate-reducing bacteria residing in the human gut as commensals, but they can produce a cytotoxic compound, hydrogen sulfide, which could have detrimental effects (Singh et al., 2023). In comparison, intermittent fasting caused limited changes to the gut microbiome and SCFAs. This could be due to the limited fasting duration as opposed to the prolonged fasting of Ramadan. Moreover, Ramadan fasting includes water restriction in addition to food. Despite the shorter period of fasting compliance in intermittent fasting, the fasting regimen was accompanied by changes in both alpha and beta diversity measures. The enrichment of family *Rikenellaceae* and some pathogenic genera such as *Peptoniphillus, Streptococcus*, and *Terrisporobacter* support this result. *Rikenellaceae* includes SCFA producing microbes, and increased abundance of this family after intermittent fasting was reported by Angoorani et al. (2021).

Differential analysis revealed temporal changes in gut microbial communities upon fasting. In intermittent fasting, an increased abundance of *Lactobacillus spp*. and *Prevotella copri* was observed; these are associated with weight loss and increased susceptibility to autoimmune diseases, respectively (Khan et al., 2022). In Ramadan, *Ruminococcaceae; CAG-352, Sarcina* abundance increased after fasting. *Ruminococcaceae; CAG-352* is an SCFA producer and is well known to have a beneficial role in human gut health due to its anti-inflammatory properties (Choi et al., 2023), whereas *Sarcina* species are residents of the upper gastrointestinal tract and are associated with gastric ulcer and delayed gastric emptying (Marcelino et al., 2021; Propst et al., 2020). After 3 months of fasting, an increase in the genera of certain opportunistic pathogens including *Enterococcus, Turibacter*, and *Klebsiella* was observed (García-Solache and Rice, 2019; Krawczyk et al., 2021). *Turibacter* has also been associated with the consumption of a high resistant starch diet in rats (García-Legorreta et al., 2020), and *Klebsiella* has been associated with high-fat-derived energy (Ali et al., 2021). This indicates that post-fasting, changes within the gut microbial communities creates a unique niche that can lead to enhanced colonization by bacteria that show a metabolic fitness advantage under simple carbohydrate-limiting conditions.

Subset analysis showed that few ASVs were observed to cause changes in beta diversity in Ramadan fasting. An increased abundance of *Prevotella* and *Collinsella* was observed after fasting contrary to Ali et al. (2021), Forslund (2023), Khan et al. (2022), and Ozkul et al. (2020). Different sample size, ethnicity, and dietary habits could be considered the important factors causing variation in these results. In the intermittent fasting group, a decreased abundance of *Faecalibacterium, Bifidobacterium*, *Roseburia, Ruminococcus_torques _group*, and *Blautia* was seen, which plays an important role in protection from metabolic and inflammatory diseases (Cheng et al., 2022; Forslund, 2023; Wang H. et al., 2023; Yan et al., 2022). *Dorea* can cause irritable bowel disease, and their decreased abundance after fasting has been observed earlier (Khan et al., 2022). Taken together, these results show that different fasting regimens impact gut microbiota differentially with few transient changes, and the gut microbiome reverts back to its pre-fasting state upon resumption of normal dietary regimens. This reversal of fasting-induced beneficial gut microbiota upon refeeding could be explained by several mechanisms. For example, fasting creates a transient niche which favors the microbes which utilize host derived substrates, such as the mucin glands. After refeeding, the rapid shift in dietary substrates favors the fast-growing carbohydrate fermenters or taxa adapted to a higher caloric intake, which in turn downregulate the relative abundance of fasting-enriched species (Ducarmon et al., 2023). During fasting, metabolic changes such as reduced insulin and altered bile acid profiles can also modulate gut microbiota through their effects on gut motility, pH, and immune regulation. Refeeding reverses these host-mediated changes, driving the gut microbiota back toward their baseline state (Jo et al., 2023; Mohr et al., 2021).

Short-chain fatty acids are produced by gut bacteria through the fermentation of dietary fiber, which impacts gut microbial composition and function (Chen et al., 2022). Although most studies report changes in major SCFA producers, the majority lack SCFA analysis. Our results suggest that fasting regimens impact certain SCFAs through a variation in gut microbiota composition and functions. The significant association of isobutyric, isopropyl hexanoic, and heptanoic acids with bacterial composition and functions in Ramadan fasting could be explained by the very high consumption of sugary products during Ramadan. Some food groups such as sweetened dairy products are already known to have a positive correlation with butyric, isobutyric, isovaleric, hexanoic, and heptanoic acid levels (Gudan et al., 2022). The majority of SCFAs, such as acetic, propionic, and butyric acids, are derived from dietary fiber, but branched SCFAs, including isobutyric and isovaleric acids, are produced by the fermentation of branched chain amino acids (valine, leucine, and isoleucine) derived from non-digestible proteins (Ríos-Covián et al., 2016). The increased branched SCFAs post-fasting could indicate lower a consumption of dietary fiber by study participants undergoing fasting and an increased proteolytic activity by the gut microbes (Wang et al., 2019). The consumption of high-fat-content food items explained the microbiome variability in composition and phylogeny after Ramadan fasting in our study. This also correlated the distribution of SCFAs within the gut with the significant metabolites isopropyl hexanoic, valeric, and heptanoic acids.

Isobutyric and isovaleric acids are branched SCFAs and are far less studied, but some studies have reported their adverse effect on gut and metabolic health due to high concentrations in the colon (Blaak et al., 2020). Murayama et al. (2024) demonstrated the strong anti-cancer properties of isobutyric acid.

Ramadan fasting showed a significant impact on the metabolic profile in comparison to intermittent fasting; this could be explained by the prolonged fasting period of Ramadan, although total calorie intake during Ramadan was not calculated. However, based on participants’ dietary patterns reported in the food frequency questionnaires, individuals tended to consume more fried items and sugary drinks during Ramadan, which suggests that total calorie reduction may not have been substantial. The increased or decreased abundance of bacterial communities and SCFAs after Ramadan fasting is associated with dietary intake. For example, *Collinsella* abundance is reduced after a high protein diet. A fiber-rich diet can also decrease the abundance of *Collinsella* and increase the growth of certain SCFA-producing bacteria which help improve metabolic health (Gomez-Arango et al., 2018). Furthermore, the increased production of SCFA such as acetic, propionic, butyric, isobutyric, and valeric acids is reported after intermittent fasting in a mouse model (Wu et al., 2022), whereas a high cholesterol diet has been found to decrease acetic, propionic, butyric, and valeric acids as well as the abundance of SCFA-producing bacteria such as *Prevotellaceae* (Liu et al., 2023). Another study reported the decreased concentration of isobutyric acid after fasting (Madkour et al., 2023). The negative correlation between bacterial communities such as *Eubacterium*, *Peptoniphilus*, and *Anaerococcus* and SCFAs such as octanoic and isobutyric acids also suggests the impact of dietary patterns and composition. For instance, octanoic acid is reported to be present in milk and coconut oil and is also used as a food additive. It are also known to be related to hepatic issues and a ketogenic diet (Cesar Filho et al., 2016). The intake of dietary fibers is positively associated with *Eubacterium* spp. and SCFA levels such as butyrate, propionate, and acetate, which have beneficial effects on inflammatory bowel disease, colorectal cancer, and metabolic syndrome (Mukherjee et al., 2020). *Peptoniphilus* and *Anaerococcus* are known as saccharolytic, butyrate-producing species (Ezaki et al., 2001). An increased production of isobutyric acid has been associated with lowered insulin and glucose concentrations in the blood (Heimann et al., 2016).

In the intermittent fasting group, few genera were observed in abundance after fasting. For example, *Libanicoccus* is a novel genus belonging to the family *Coriobacteriaceae*, but their role in human physiology is not yet well studied (Bilen et al., 2018). The *Lachnospiraceae* family is the most abundant, and major SCFAs such as the butyrate-producing family of the phylum *Firmicutes* play important roles in maintaining the host’s physiological homeostasis by providing nutritional energy to the epithelial compartment (Su et al., 2022). *Peptoniphilus* is also a butyrate-producing genus, and its high abundance is associated with a red-meat-rich diet (Bodykevich et al., 2023). These findings are consistent with previous reports demonstrating that IF affect the host’s metabolic health by increasing the abundance of butyrate and propionate producing microbes (Pramono et al., 2024).

## Conclusion

Our findings suggest that Ramadan fasting is significantly associated with an increased abundance of health-promoting and SCFA-producing bacteria. However, these changes were reversed after normal dietary resumption. While most SCFA producer taxa reverted post fasting, we observed sustained increases in certain Enterobacteriaceae (*Enterococcus and Klebsiella*) at T3, indicating lasting niche changes that merit further study. Shifts in metabolic profile also highlight the potential benefits of dietary intervention in human health. Intermittent fasting showed a limited variation in gut microbiome due to the shorter fasting period and needs further investigation. To sustain fasting-induced changes after a normal feeding routine, several dietary modifications consisting of high-fiber, prebiotic-rich foods and polyphenols may help maintain the level of beneficial microbes. In addition, targeted probiotic or synbiotic supplementation could also support the resilience of beneficial taxa during dietary shifts, although controlled trials are needed to confirm efficacy.

## Data Availability

The dataset presented in this study is available under ENA repository, accession number PRJEB59240.

## Abbreviations

ASV, amplicon sequence variant; AJK, Azad Jammu and Kashmir; BMI, body-mass index; CUI, COMSATS University Islamabad; DIABLO, Data Integration Analysis for Biomarker discovery using Latent variable approaches for Omics; ICT, Islamabad Capital Territory; KPK, Khyber Pakhtunkhwa; QCAT, Quasi-Conditional Association Test; rRNA, ribosomal ribonucleic acid; SCFA, short-chain fatty acid.
