# Supplementary material for: Dietary composition and fasting regimens differentially impact the gut microbiome and short-chain fatty acid profile in a Pakistani cohort
Source: Front Syst Biol. 2025 Oct 17;5:1622753. doi: 10.3389/fsysb.2025.1622753 (PMC12575385; doi:10.3389/fsysb.2025.1622753)
Supplement: Supplementary file 1 [file Supplementaryfile1.docx]

Supplementary Material

1. **Statistical Methods**

The R’s vegan package (Oksanen et al., 2013) was used for alpha and beta diversity analyses.

For alpha diversity measures we used: (i) *Shannon entropy* – a commonly used index to measure balance within a community; (ii) *rarefied richness* – the estimated number of species/features in a rarefied sample (to minimum library size). It is also exponential of Shannon entropy; and (iii) *Simpson’s Index* – similar to Shannon entropy however the value of this index ranges between 0 and 1, with greater the value, the greater the sample diversity. It also represents the probability that two microbes randomly selected from a sample will belong to different species.

Null modeling technique was applied to gain quantitative insights into the underlying ecological mechanisms, whether the microbial community assembly is stochastic (driven by competition among taxa as a result of competitive exclusion) or deterministic (driven by environmental pressures in the host environment). Two metrics were used in this regard: a Nearest Taxon Index (NTI) employed using ses.mntd() function (local measure of phylogenetic clustering), and the Nearest Relative Index (NRI) employed using ses.mpd() function (global measure of phylogenetic clustering) from the R’s picante (Kembel et al., 2010) package, respectively. Note that NTI and NRI represent the negative of the output from ses.mntd()and ses.mpd()functions, respectively. They also quantify the number of standard deviations that the observed Mean Nearest Taxon Distance (MNTD)/ Mean Phylogenetic Distance (MPD) is from the mean of the null distribution (999 randomization by using null.model = “richness” in the ses.mntd()and ses.mpd()functions and by only considering the incidence data, i.e., the taxa are considered as present or absent). Calculation of these measures are based on the recommendations given by Stegen and colleagues (Stegen et al., 2012).

Local Contribution to Beta Diversity (LCBD) (Legendre & De Cáceres, 2013), a one-dimensional version of beta diversity was performed using the
LCBD.comp() function from the R’s adespatial package (Dray et al., 2018). LCBD dives the total beta diversity across all samples by giving them each a proportion. This way, it provides a mean to assess if an individual sample stands out with its beta diversity deviating from the average beta diversity of all the samples. For LCBD calculation, different beta diversity distance measures were used: Bray-Curtis distance; Unweighted UniFrac distance; Weighted UniFrac distance; and Hierarchical Meta-Storms (HMS) (Yufeng Zhang et al., 2021). HMS is a preferred beta diversity metric over Bray-Curtis distance for KEGG Ortholog (KOs) abundance table as it measures the beta diversity distance of functions in a hierarchical fashion by distributing the KOs abundances upward to the pathways they are part of by utilising KEGG BRITE hierarchy. R's aov()function was used to calculate the pair-wise analysis of variance (ANOVA), with the representation of p-values drawn on top of alpha diversity and LCBD figures. To adjust for the paired-nature of the samples, i.e. coming from the same subjects, all the pairwise statistics were done using *One-way within ANOVA* (http://www.cookbook-r.com/Statistical_analysis/ANOVA/) as aov(value ~ Groups + Error(SubjectID/Groups)) with samples coming from the same subjects connected with lines. The beta diversity was plotted using Principal Coordinate Analysis (PCoA) from the R’ Vegan’s package. PERMANOVA analyses was additionally performed to get the percentage variability explained in the beta diversity against the sources of variation (covariates considered in this study).

To find the core microbiome, genera (ASVs collated at genus level) with minimum prevalence of 50% across all samples were identified. In this regard, R’s microbiome package (Lahti & Shetty, 2017) is used to obtain two-dimensional plots of core microbiome with the first dimension being different detection thresholds of reads (increasing in number) at which the prevalence across samples is established. In the second dimension, these genera were then sorted in terms of overall mean prevalence giving ranking of core genera in terms of increasing abundance (from left to right changing from low abundant core genera to high abundant core genera).

For Ramadan fasting and intermittent fasting, same individuals provided multiple samples across the course of study, and therefore a specialised cluster association test (Tang & Chen, 2021) utilising the R’s miLineage package (https://tangzheng1.github.io/tanglab/software.html) was used. This test is referred to as QCAT-C test and is robust against complex correlations that arise due to the paired nature of samples. We have used the QCAT_GEE.Cluster() function (with default values) from the R’s miLineage package. QCAT-C test fits two separate models to microbes, those that have excessive zero values, and those that do not have (referred to as positive microbes). Furthermore, the test simultaneously gives differential taxa at different lineages (Phylum, Class, Order, Family, and Genus). For visualisation purposes, the abundances of differential taxa (at different taxonomic ranks) were normalised using *Total Sum Scaling* was used followed by a *Centralized Log Ratio* (TSS+CLR) transformation.

We used the “BVSTEP” routine (Clarke & Ainsworth, 1993), an algorithm that searches for highest correlation (Mantel test) between two abundance tables, one with fixed number of features, and one with variable number of features, that can be permuted. Taking the second table as the copy of the first table then implodes the abundance table down to the absolute minimal set of features (ASVs in our case) that roughly conserve the same beta diversity distance between the samples as the full set of features, thus we are only left with the main patterns that change between multiple categories (time points in our case). For this purpose, we have used bvStep() from R’s sinkr package (Taylor, 2014).This analysis is complimentary to the differential analysis and identified the ASVs that were causing the major shifts in beta diversity.

To find genera that are at least 2 log2 fold different (adjusted p-value < 0.05) between multiple conditions, DESeqDataSetFromMatrix()function from the DESeq2 package (Love et al., 2014) was used. This function is used to acquire the maximum likelihood estimates for log fold change of genera between the two conditions by using a negative binomial Generalised Linear Model (GLM). The test then employs Bayesian shrinkage to obtain shrunken log fold changes subsequently applying the Wald test to establish significances.

In addition to the fecal samples, the participants also provided filled questionnaires about their life and eating styles (Supplementary Questionnaire). On this data, which was primarily categorical in nature, we have applied $\chi^{2}$ test (<http://www.sthda.com/english/wiki/chi-square-test-of-independence-in-r>) to see if there is inherent dependency between some of the questions, by representing the data in the form of a contingency table. Once the nature of dependency was established, we then plotted $\chi^{2}$ contribution diagram to rank the categories (cells of contingency table) that contributed most to the relationship. We also drew pearson residuals with positive residuals shown in blue. Positive values in cells specify an attraction (positive association) between the corresponding row and column variables of the contingency table whilst negative residuals are shown in red. This implies a repulsion (negative association) between the corresponding row and column variables. To further explore the incidence rate ratio, we then fitted generalized linear model GLM on the count of contingency table as Freq ~ Var1 + Var2 in glm() using Poisson distribution as a family.

To select the SCFAs/other parameters (including average food intake frequency during Ramadan) most strongly associated with the variance of the observed communities for both Intermittent fasting and Ramadan fasting samples, redundancy analysis (RDA) was applied on different beta diversity distances using Vegan’s capscale() and ordistep() functions in the following set of commands: cap.env = capscale( abund_table.dist~., meta_table); mod0.env = capscale( abund_table.dist~1, meta_table); step.env=ordistep(mod0.env, scope=formula(cap.env), direction=”both”, Pin=0.1, perm.max=9999, R2scope=TRUE). step.env$anova with p<0.05 was then able to identify the subset of SCFAs which were later used in the PERMANOVA analysis using adonis2() function from R’s vegan package. This approach was previously used in (Vass et al., 2020) as a variable selection approach before applying PERMANOVA analysis. For abund_table.dist, different beta diversity distances including Bray-Curtis, Unweighted UniFrac, Weighted UniFrac, and Hierarchical Meta-Storms were used. For drawing the density of food intake frequency during Ramadan, R’s ggridges package (Wilke, 2021) was used.

We have used DIABLO algorithm from R’s mixOmics package (Rohart et al., 2017) to integrate M=3 datasets denoted by $X^{\left( 1 \right)}\left( N\times P_{1} \right)$, $X^{\left( 2 \right)}\left( N\times P_{2} \right)$, $X^{\left( 3 \right)}\left( N\times P_{3} \right)$ where $X^{\left( 1 \right)}$ represents the TSS+CLR (Total Sum Scaling + Centralised Log Ratio) normalised ASVs abundance table, $X^{\left( 2 \right)}$represents the autoscaled SCFA table, and $X^{\left( 3 \right)}$ denotes the dummified outcome matrix (i.e., binary representation of labelling such as IB_T1 and IF_T2 for Intermittent Fasting samples, and RB_T1, RF_T2, and RF_T3 for Ramadan Fasting samples). The DIABLO algorithm then factorizes each of these datasets into scores and loading vectors (also called principal components) in such a way that the covariance of the scores between these datasets are maximized. For $q=1,2,\ldots,Q,$ datasets, DIABLO solves for each principal component $h=1,\ldots,H$

$$\begin{matrix} \mathbf{arg max} \\ a_{h}^{(1)},\ldots,a_{h}^{(Q)} \end{matrix} \sum_{q,j=1,q\neq j}^{Q} c_{q,j} \mathrm{cov}\left( X_{h}^{(q)}a_{h}^{(q)},X_{h}^{(j)}a_{h}^{(j)} \right) s.t. \left\| a_{h}^{(q)} \right\|_{2}=1 \mathrm{and}\left\| a_{h}^{(q)} \right\|_{1}\leq\lambda^{(q)}$$

Where $a_{h}^{(q)}$ is the loading vector on component h associated with the matrix $X_{h}^{(q)}$ of the data set $X^{(q)}$. $C=\left\{ c_{q,j} \right\}_{q,j}$ is the design matrix. $C$ is a $Q\times Q$ matrix that specifies whether datasets should be correlated and includes values between 0 and 1. According to recommendations given by R’s mixOmics, a full weighted design is incorporated where $c_{q,j}=0$.1 between data matrices $X^{\left( 1 \right)}$ and $X^{\left( 2 \right)}$ and $c_{q,j}=1$ where the outcome matrix is involved (between $X^{\left( 1 \right)}$ and $X^{\left( 3 \right)}$ and between $X^{\left( 2 \right)}$ and $X^{\left( 3 \right)}$). This leads to a reasonable trade-off between maximizing correlation between datasets and finding the discriminant features across the outcome (IB_T1 and IF_T2 for Intermittent Fasting samples, and RB_T1, RF_T2, and RF_T3 for Ramadan Fasting samples). Since the coefficients of the loading vectors serves as a weight for individual features (microbes or SCFAs) to calculate the scores, an additional $l_{1}$ penalty constraint $\left\| a_{h}^{(q)} \right\|_{1}\leq\lambda^{(q)}$ was incorporated in the optimization function. Here, $\lambda^{(q)}$ is the penalization parameter in the $l_{1}$ penalty constraint ensuring some of the coefficients of the loading components to go to zero, with non-zero coefficients giving the discriminating features (microbes or SCFAs). The other constraint $\left\| a_{h}^{(q)} \right\|_{2}=1$ ensures that the loading vector have a unit magnitude.

To predict the number of loading vectors (principal components), and then the number of non-zero coefficients of these loading vectors, block.splsda() and tune.block.splsda()functions from R’s mixOmics package were used. For both cases (Intermittent and Ramadan fasting), the optimisation process involves: a) fine tuning the model using leave-one-out cross-validation by splitting the data into training and the testing sets, and then identifying the number of loading components (principal components) that maximise the class separation using any of the distance measures between samples that lie across different region boundaries identified for classes; and b) finding the non-zero coefficients for each of the loading components. For Intermittent fasting samples, ncomp=2 components and dist=”centroids.dist” was used, whilst for Ramadan fasting, ncomp=3 components and dist=”mahalanobis.dist” was used in the tune.block.splsda() function, respectively.

To visualise the correlation between SCFAs and average food frequency intake, R’s ComplexHeatmap package (Gu et al., 2016) was used after calculating significances using cor.test() with pearson correlation method.

**Supplementary Table 1**: Calculated SCFA level in different time points of intermittent and Ramadan fasting groups.

| Sample_ID | Treatment | C2 | C3 | IC4 | C4 | IC5 | C5 | IC6 | C6 | C7 | C8 | Total |
| --- | --- | --- | --- | --- | --- | --- | --- | --- | --- | --- | --- | --- |
| IF1_1 | Intermittent_fasting | 222.9726 | 4.413704 | 1.59272 | 2.945076 | 3.797044 | 1.067424 | 0.43488 | 1.292777 | 0.419131 | 0.531728 | 239.4671 |
| IF1_2 | Intermittent_fasting | 109.46 | 1.590605 | 0.621926 | 1.757509 | 0.962842 | 0.662297 | 1.628918 | 1.364475 | 0.107692 | 0.112079 | 118.2684 |
| IF2_1 | Intermittent_fasting | 190.8694 | 8.785899 | 11.45898 | 5.501761 | 11.24188 | 0.474262 | 1.443563 | 1.277028 | 0.079122 | 0.154988 | 231.2869 |
| IF2_2 | Intermittent_fasting | 512.3617 | 125.996 | 10.32322 | 136.2093 | 15.21229 | 22.20343 | 0.421978 | 11.86804 | 1.499728 | 0.148804 | 836.2445 |
| IF3_1 | Intermittent_fasting | 913.7939 | 315.6678 | 16.773 | 470.4175 | 23.19087 | 39.78239 | 0.804271 | 40.37619 | 2.85369 | 1.700379 | 1825.36 |
| IF3_2 | Intermittent_fasting | 198.5851 | 4.772054 | 3.713014 | 6.107283 | 6.030634 | 0.937712 | 1.456282 | 1.063968 | 0 | 0 | 222.666 |
| IF4_1 | Intermittent_fasting | 682.1898 | 99.43183 | 13.37031 | 192.3111 | 24.18028 | 52.41253 | 1.217623 | 7.835883 | 0.124713 | 0.417431 | 1073.492 |
| IF4_2 | Intermittent_fasting | 398.1044 | 67.28052 | 11.27521 | 48.38085 | 18.03294 | 11.48456 | 0.394327 | 1.327642 | 0.087488 | 0.141885 | 556.5098 |
| IF5_1 | Intermittent_fasting | 153.4151 | 2.429407 | 7.479353 | 1.414044 | 2.303823 | 0.656019 | 0.300347 | 0.525222 | 0.17314 | 0.559283 | 169.2558 |
| IF5_2 | Intermittent_fasting | 1845.649 | 311.5038 | 46.07477 | 235.2058 | 65.90215 | 60.78042 | 0.89739 | 6.070588 | 0.103063 | 0.269364 | 2572.457 |
| IF7_1 | Intermittent_fasting | 325.919 | 37.20253 | 9.703644 | 15.41444 | 13.87852 | 5.870604 | 0.490352 | 1.314161 | 0 | 1.941909 | 411.7352 |
| IF7_2 | Intermittent_fasting | 551.7543 | 95.67031 | 14.5728 | 49.39804 | 17.86134 | 14.90605 | 0.318189 | 3.895893 | 0.526258 | 0.114883 | 749.018 |
| IF8_1 | Intermittent_fasting | 895.8611 | 332.3934 | 22.73639 | 299.2742 | 35.55888 | 99.06362 | 0.6337 | 24.29075 | 3.411721 | 0.855203 | 1714.079 |
| IF8_2 | Intermittent_fasting | 933.7538 | 377.6516 | 20.50922 | 248.6107 | 26.31967 | 59.88331 | 0.698915 | 2.674932 | 0 | 0.243679 | 1670.346 |
| IF10_1 | Intermittent_fasting | 118.5948 | 3.843019 | 1.433404 | 1.547387 | 2.928415 | 1.289285 | 0.215221 | 0.619844 | 0 | 0.178365 | 130.6498 |
| IF10_2 | Intermittent_fasting | 223.5865 | 6.290582 | 25.6444 | 14.03552 | 20.11358 | 1.231925 | 1.814364 | 1.278086 | 0.135316 | 0.049938 | 294.1802 |
| IF11_1 | Intermittent_fasting | 134.1989 | 3.480514 | 0.918151 | 2.46913 | 2.045651 | 1.817241 | 0.347507 | 0.744315 | 0 | 0 | 146.0214 |
| IF11_2 | Intermittent_fasting | 144.5489 | 3.263843 | 1.245313 | 1.70269 | 3.003519 | 1.649143 | 0.422575 | 1.192265 | 0.056796 | 0.256642 | 157.3417 |
| RF1_1 | Ramadan_fasting | 1500.28 | 828.7661 | 4.519778 | 609.4812 | 4.273767 | 27.17229 | 1.582573 | 12.3991 | 0.763326 | 0.571869 | 2989.81 |
| RF1_2 | Ramadan_fasting | 845.3852 | 230.5205 | 10.80367 | 132.3389 | 16.01884 | 54.54925 | 0.594873 | 4.523968 | 0.764206 | 3.036869 | 1298.536 |
| RF1_3 | Ramadan_fasting | 1198.22 | 315.0226 | 10.96094 | 274.4525 | 12.22801 | 26.1063 | 2.741153 | 5.814955 | 0.336663 | 0.16607 | 1846.049 |
| RF2_1 | Ramadan_fasting | 853.7138 | 248.0287 | 27.91907 | 333.2718 | 41.39301 | 88.31477 | 0.881339 | 32.51937 | 4.952923 | 0.428366 | 1631.423 |
| RF2_2 | Ramadan_fasting | 1024.201 | 436.5646 | 48.92819 | 560.8617 | 62.73122 | 61.33046 | 2.866464 | 27.45099 | 2.399154 | 0.086555 | 2227.42 |
| RF2_3 | Ramadan_fasting | 778.5508 | 296.2242 | 20.55674 | 268.5785 | 32.22532 | 88.7079 | 0.633502 | 21.71187 | 3.343213 | 0.339617 | 1510.872 |
| RF3_1 | Ramadan_fasting | 495.5934 | 152.4494 | 0.978346 | 109.958 | 1.552356 | 26.17708 | 0.350291 | 1.568547 | 0.158361 | 0.989761 | 789.7756 |
| RF3_2 | Ramadan_fasting | 598.3973 | 130.0657 | 15.15009 | 150.1247 | 19.32841 | 25.32707 | 0.874264 | 16.94721 | 2.224005 | 0.215779 | 958.6544 |
| RF3_3 | Ramadan_fasting | 805.0056 | 381.0557 | 31.4135 | 355.1704 | 48.14174 | 202.7145 | 0.532671 | 18.83988 | 2.265865 | 1.143225 | 1846.283 |
| RF4_1 | Ramadan_fasting | 1212.172 | 377.3606 | 4.712524 | 536.1627 | 5.760589 | 24.59055 | 2.064813 | 9.791207 | 0.456636 | 3.136098 | 2176.208 |
| RF4_2 | Ramadan_fasting | 1492.591 | 459.3052 | 22.72087 | 463.7491 | 27.95938 | 59.66388 | 3.242802 | 34.6568 | 3.378527 | 0.257522 | 2567.525 |
| RF4_3 | Ramadan_fasting | 249.864 | 6.635614 | 1.552051 | 3.412373 | 6.464011 | 0.652307 | 0.500183 | 1.201961 | 0.067932 | 0 | 270.3504 |
| RF5_1 | Ramadan_fasting | 78.58699 | 2.364527 | 3.62928 | 2.050063 | 7.57841 | 0.970548 | 0.467692 | 0.671867 | 0.209177 | 0.064115 | 96.59267 |
| RF5_2 | Ramadan_fasting | 323.0015 | 44.95376 | 75.44954 | 100.0153 | 146.1095 | 44.96868 | 22.03976 | 12.40421 | 1.09328 | 0.31391 | 770.3494 |
| RF5_3 | Ramadan_fasting | 333.8389 | 12.35641 | 73.40608 | 88.92928 | 116.7312 | 35.53152 | 28.52854 | 15.39671 | 0.456733 | 0.216681 | 705.392 |
| RF7_1 | Ramadan_fasting | 27.79309 | 0.928269 | 0.480321 | 3.656084 | 1.155226 | 0.683561 | 0.417248 | 0.717317 | 0 | 0 | 35.83111 |
| RF7_2 | Ramadan_fasting | 1217.405 | 242.9573 | 22.13667 | 300.2852 | 30.0908 | 36.35996 | 0.877896 | 15.74231 | 1.812691 | 12.21599 | 1879.884 |
| RF7_3 | Ramadan_fasting | 146.653 | 13.81092 | 1.006438 | 17.28407 | 1.417621 | 2.43045 | 0.59355 | 2.643286 | 0.214798 | 0.112692 | 186.1668 |
| RF8_1 | Ramadan_fasting | 204.7332 | 5.200094 | 67.35314 | 12.78052 | 85.07634 | 2.703332 | 2.545286 | 1.278924 | 0.093823 | 1.880469 | 383.6451 |
| RF8_2 | Ramadan_fasting | 1202.016 | 268.7039 | 121.2273 | 640.3595 | 179.5793 | 105.2565 | 10.77306 | 16.27559 | 0.076036 | 8.916972 | 2553.185 |
| RF8_3 | Ramadan_fasting | 85.24761 | 8.230197 | 4.746169 | 4.231695 | 7.825115 | 0.550904 | 0.352922 | 0.603054 | 0.086894 | 0.071022 | 111.9456 |
| RF9_1 | Ramadan_fasting | 155.4538 | 3.592073 | 0.563061 | 1.725472 | 0.921651 | 2.14174 | 0.506958 | 1.724049 | 0.132866 | 1.01858 | 167.7802 |
| RF9_2 | Ramadan_fasting | 289.6767 | 415.3644 | 44.16355 | 161.8117 | 20.31456 | 5.206015 | 3.472093 | 2.992867 | 0 | 0 | 943.0019 |
| RF9_3 | Ramadan_fasting | 1117.419 | 446.4898 | 23.97085 | 283.7711 | 29.19084 | 64.04745 | 0.95845 | 2.503895 | 0.218162 | 2.457778 | 1971.027 |
| RF10_1 | Ramadan_fasting | 2027.15 | 560.0305 | 51.4612 | 439.9333 | 70.57206 | 130.5074 | 0.800311 | 16.71357 | 2.061494 | 1.124925 | 3300.355 |
| RF10_2 | Ramadan_fasting | 242.206 | 8.267491 | 1.989312 | 5.234504 | 3.363604 | 1.985237 | 0.519166 | 1.263831 | 0.16209 | 0.114671 | 265.1059 |
| RF10_3 | Ramadan_fasting | 144.57 | 3.023871 | 0.707164 | 1.538761 | 2.012552 | 3.401086 | 0.404833 | 0.77993 | 0.360887 | 0.082361 | 156.8814 |

**Supplementary Questionnaire**


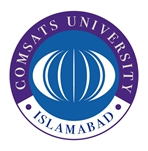


# Pakistan Microbiome Initiative

1. **OFFICIAL USE ONLY**

**Inspection Date and Time:**

**Gut Kit ID:**

***Note: You may decline to answer any question.***

# BIOGRAPHICAL DATA

Name: *E*-mail:

Phone no: Gender:

Age: Marital Status:

No. of family members living in the household:

City of Birth: Current City:

Ethnicity: Caste:

# VITAL SIGNS

| Height (*in*): | Weight (*kg*): | BMI (*kg/m^2^*): |
| --- | --- | --- |
| Blood Glucose (*mg/dl*): | Pulse Rate (*per min*): |  |
| Temperature (*Fº*): | Blood Pressure (*mmHg*): |  |

1. **EXCLUSION CHECKLIST (If you answer ‘Yes’ to any question from 1-17, please do not proceed with the questionnaire).**

| **#** | **Question** | **Yes** | **No** |
| --- | --- | --- | --- |
| 1 | Are you aged below 18? |  |  |
| 2 | Is your BMI <18 or >30 Kg/m^2^? |  |  |
| 3 | Have you taken any antibiotics in the last 3 months? |  |  |
| 4 | Have you taken any multivitamins or dietary supplements in the last 1 month? |  |  |

| 5 | Do you have (or had) acute or chronic diarrhea in the last 2 months? |  |  |
| --- | --- | --- | --- |
| 6 | Do you have history of hypertension, colon cancer, or inflammatory bowel disease? |  |  |
| 7 | Are you lactose or gluten intolerant? |  |  |
| 8 | Are you allergic to any food? |  |  |
| 9 | Do you have irregular menstrual cycles (that is less than 21 or more than 35 days apart)? |  |  |
| 10 | Are you pregnant or lactating? |  |  |
| 11 | Have you been vaccinated for any disease in the last 3 months? |  |  |
| 12 | Do you currently have urinary tract infection? |  |  |
| 13 | Do you consume any recreational drugs like smoking, marijuana? |  |  |
| 14 | Have you travelled internationally in the last 6 months? |  |  |
| 15 | Have you had bloody stools, constipation, dizziness, or fever in the last 14 days? |  |  |
| 16 | Did you contract COVID-19 in the last 3-6 months? |  |  |
| 17 | Have you been diagnosed with any chronic (that lasts one year or more) medical or dental condition(s)? |  |  |

# PRE-RAMADAN DIETARY HABITS

| **#** | **Question** | **Yes** | **No** | **N/A** | **No answer** |
| --- | --- | --- | --- | --- | --- |
| **1** | Did you eat breakfast on at least 5 days each week? |  |  |  |  |
| **2** | Did you eat lunch on at least 5 days each week? |  |  |  |  |
| **3** | Did you eat dinner on at least 5 days each week? |  |  |  |  |
| **4** | Did you consume snacks in between meals? |  |  |  |  |
| **5** | Did you consume honey regularly? |  |  |  |  |
| **6** | Did you eat goat and/or lamb meat regularly? |  |  |  |  |
| **7** | Did you eat chicken regularly? |  |  |  |  |
| **8** | Did you eat fish regularly? |  |  |  |  |
| **9** | Did you eat beef regularly? |  |  |  |  |
| **10** | Did you eat rice regularly? |  |  |  |  |
| **11** | Did you eat naan/roti on at least 5 days each week? |  |  |  |  |
| **12** | Did you eat vegetables regularly? |  |  |  |  |
| **13** | Did you consume “junk food” (e.g. fried chicken burgers,  pizza) at least twice every week? |  |  |  |  |
| **14** | Did you consume any of the following probiotics every day? Please tick mark.   - Yogurt - Pickles - Soft cheese - Buttermilk |  |  |  |  |
| **15** | Did you consume any of the following prebiotics every day? Please tick mark.   - Maple syrup - Dark chocolate - Asparagus - Oatmeal - Legumes |  |  |  |  |
| **16** | Did you consume eggs every day? |  |  |  |  |
| **17** | Did you consume following dairy products every day? please tick mark.   - Bread - Milk - Butter - Cream - Lassi - Cheese - Desi ghee |  |  |  |  |
| **18** | Did you eat fresh fruits every day? |  |  |  |  |
| **19** | Did you consume dry fruits 2 or more times a week? |  |  |  |  |
| **20** | Did you smoke regularly? |  |  |  |  |
| **21** | Did a family member or friend you are in constant contact with (>2 hours a day) smoke in your presence? |  |  |  |  |
| **22** | Did you consume alcohol regularly? |  |  |  |  |
| **23** | Did you consume tea every day? |  |  |  |  |
| **24** | How many cups of tea did you consume per day?   - 0 - 1 - 2 - 3 - More than 3 |  |  |  |  |
| **25** | Did you consume coffee every day? |  |  |  |  |
| **26** | How many cups of coffee you consume per day?   - 0 - 1 - 2 - 3 - More than 3 |  |  |  |  |

# How often did you eat dinner outside instead of eating/cooking at home?

(a) Once a week (b) Between 2-4 times a week

(c) Everyday (d) Rarely

# What best described your diet?

1. Vegetarian only and no meat
2. No beef and no goat but consume chicken/fish
3. All kinds of meat and vegetables
4. Eat vegetables and meat but no dairy products

# How often did you eat Samosa, pakora and other deep-fried food items in a week?

(a) Once a week (b) Between 2-4 times a week

(c) Everyday (d) Rarely

# How often did you eat sugary foods (e.g. halwa, desserts, kheer, ice-cream, etc.) in a week?

(a) Once a week (b) Between 2-4 times a week

(c) Everyday (d) Rarely

# How often did you take juices or other sugary drinks (e.g. lemonade, Rooh Afza, soft drinks, etc.) in a week?

(a) Once a week (b) Between 2-4 times a week

(c) Everyday (d) Rarely

# DIETARY HABITS DURING RAMADAN

**(To be filled DURING Ramadan)**

| **#** | **Question** | **Yes** | **No** | **N/A** | **No answer** |
| --- | --- | --- | --- | --- | --- |
| **1** | Did you eat Sehri regulary (i.e., >5 times a week)? |  |  |  |  |
| **2** | Did you eat Iftar regularly (i.e., >5 times a week)? |  |  |  |  |
| **3** | Did you eat dinner after Iftari (i.e., >5 times a week)? |  |  |  |  |

# How often did you eat Samosa, pakora and other deep-fried food items during Ramadan?

(a) Once a week (b) Between 2-4 times a week

(c) Everyday (d) Rarely

# How often did you eat sugary foods (e.g. halwa, desserts, kheer, ice-cream, etc.) during Ramadan?

(a) Once a week (b) Between 2-4 times a week

(c) Everyday (d) Rarely

# How often did you eat dairy products (e.g. bread, milk, cream, cheese, lassi, butter, desi ghee etc.) during Ramadan?

(a) Once a week (b) Between 2-4 times a week

(c) Everyday (d) Rarely

# How often did you consume probiotics (e.g. yogurt, pickle, soft cheese, buttermilk, etc.) during Ramadan?

(a) Once a week (b) Between 2-4 times a week

(c) Everyday (d) Rarely

# How often did you consume prebiotics (e.g. dark chocolate, asparagus, legumes, etc.) in Ramadan?

(a) Once a week (b) Between 2-4 times a week

(c) Everyday (d) Rarely

# How often did you take juices or other sugary drinks (e.g. lemonade, rooh afza, soft drinks, etc.) in Ramadan?

(a) Once a week (b) Between 2-4 times a week

(c) Everyday (d) Rarely

# What best described your diet during Ramadan?

1. Vegetarian only and no meat
2. No beef and no goat but consume chicken/fish
3. All kinds of meat and vegetables
4. Eat vegetables and meat but no dairy products

**(To be filled AFTER Ramadan)**

| **#** | **Question** | **Yes** | **No** | **N/A** | **No answer** |
| --- | --- | --- | --- | --- | --- |
| **1** | Do you take breakfast regularly after Ramadan? |  |  |  |  |
| **2** | Do you take lunch regularly after Ramadan? |  |  |  |  |
| **3** | Do you take dinner regularly after Ramadan? |  |  |  |  |

**4. How many fasts did you skip?**

|  |
| --- |

1. **Whether they were continuous or random interruptions?**

|  |
| --- |

1. **Did you keep additional fasts after Eid?**

|  |
| --- |

1. **Did you observe any changes in your sleep pattern?**

|  |
| --- |

1. **Did you find it difficult to be motivated for work during and after Ramadan?**

|  |
| --- |

1. **Did you lose /gain weight during Ramadan? If yes, please mention how many Kgs You lose/gain.**

|  |
| --- |

**9. How many times you did ablution (Wuzu) daily in Ramadan?**

(a) 5 times a day (b) 3 times a day

(c) 2 times a day (d) Once every day

(e) None of the above

# MEDICAL HEALTH QUESTIONAIRE

| **#** | **Question** | **Yes** | **No** | **N/A** | **No answer** |
| --- | --- | --- | --- | --- | --- |
| **1** | Did you get your appendix removed? |  |  |  |  |
| **2** | Do you experience acid reflux? |  |  |  |  |
| **3** | Do you experience gut flare-ups (Sudden pain, or outburst in gut)? |  |  |  |  |
| **4** | Have you had your colon cleansed in the past week? |  |  |  |  |
| **5** | Have you ever used any medication or home remedies to prevent or treat constipation? Please mention if known. |  |  |  |  |
| **6** | Do you experience lethargy or general malaise? |  |  |  |  |
| **7** | Did you suffer from parasitic infection (tapeworm etc.) during childhood? |  |  |  |  |
| **8** | If so, had you undergone any treatment for parasitic infection? |  |  |  |  |
| **9** | Did you suffer from anemia in the past? |  |  |  |  |
| **10** | Did you have any craving for soil/chalk/sand/ or other non-food items in the past? |  |  |  |  |
| **11** | Are you allergic to any of the following food items? If yes, please tick mark   - Soybeans. - Peanuts. - Milk. - Wheat. - Eggs. - Fish (bass, flounder and cod) - Shellfish (crab, crayfish, lobster and shrimp) - Tree nuts (almonds, walnuts and pecans) |  |  |  |  |
| **12** | Do you have any of the following problems?   - Celiac disease - Lactose sensitivity |  |  |  |  |
| **13** | Were you given antibiotics as a child? |  |  |  |  |
| **14** | Were you breastfed as an infant? |  |  |  |  |
| **15** | What was your mode of birth (C-section or natural), if known. |  |  |  |  |
| **16** | Do you have trouble biting or chewing certain foods (apples, meat)? |  |  |  |  |
| **17** | Do you have trouble swallowing? |  |  |  |  |
| **18** | Have you had your tonsils removed? |  |  |  |  |
| **19** | Do you regularly experience throat issues (e.g. sore throat, flu? |  |  |  |  |
| **20** | Is your mouth dry? |  |  |  |  |
| **21** | Do you have bad breath? |  |  |  |  |

1. **GENERAL QUESTIONS**

| **#** | **Question** | **Yes** | **No** | **N/A** | **No answer** |
| --- | --- | --- | --- | --- | --- |
| **1** | Do you exercise regularly? |  |  |  |  |
| **2** | Do you have children? |  |  |  |  |
| **3** | Do you have pets? |  |  |  |  |
| **4** | Do you have contact with livestock? |  |  |  |  |
| **5** | Do you go to the dentist at least once a year? |  |  |  |  |
| **6** | Do you share your toothbrush with anyone? |  |  |  |  |
| **7** | Do you share your bathroom/toilet? |  |  |  |  |
| **8** | Do you feel you are becoming healthier? |  |  |  |  |
| **9** | Do you feel you are becoming unhealthier? |  |  |  |  |

1. **How often do you poop?**

(a) Once in 2-3 days (b) Once every day (c) 2-3 times a day

# 11. How do you feel during bowel movement?

(a) Good or very good (b) Moderate (c) Poor or painful

# 12. Have you felt abdominal pain in the last 2 days?

(a) Yes-extreme (b) Yes-moderate

(c) Yes-Slight (d) No-not recently

# 13. What is your main source of drinking water?

(a) Tap (b) Filtered

(c) Bottled (d) Mineral

# 14. Before Ramadan, did you have any of the following symptoms?

(a) Bloody stools (b) Constipation (c) Dizziness

(d) Fever (e) Diarrhea (f) Insomnia

(g) Migraine (h) Stomach pain (i) Rash

(j) Headache

# 15. After Ramadan, did you have any of the following symptoms?

(a) Bloody stools (b) Constipation (c) Dizziness

(d) Fever (e) Diarrhea (f) Insomnia

(g) Migraine (h) Stomach pain (i) Rash

(j) Headache

# 16. How often do you get sick?

(a) Never (b) Rarely (c) Sometimes

(d) Frequently (e) All the time

# 17. What best describes your employment status?

(a) Full-time (b) Self-employed (c) Part-time

(d) Unemployed (e) Student (f) Retired

# 18. How many hours per week do you work?

(a) Less than 20 (b) 20-40

(c) 40-60 (d) more than 60

# What best describes your relationship status?

(a) Single (b) Married (c) In a relationship/Engaged

(d) Separated/Divorced (e) Widowed

# How do you identify yourself as a person?

(a) Very social and friendly (b) Sometimes social and sometimes friendly

(c) Shy and introvert (d) Social only with very close friends

(e) Stressed at the prospect of social gatherings

# What is your occupation (or occupation of parent/legal guardian)?

(a) Academia (b) Labor force

(c) Military (d) Personal business

(e) Agriculture (f) Unemployed

# What is your monthly income (or income of parent/guardian)?

(a) <20,000 PKR (b) Between 20,000 and 60,000 PKR

(c) Between 60,000 and 120,000 PKR (d) >120,000 PKR

1. **What best describes your socioeconomic status?**

(a) Lower class (b) Lower middle class (c) Middle class

(d) Upper middle class (e) Upper Class

**24. What is the highest level of education you completed?**

(a) Did not go to school (b) Matriculation (c) Intermediate

(d) Bachelor degree (e) Masters (f) Doctorate

**25. In general, how would you describe your health?**

(a) Excellent (b) Good (c) Moderately healthy

(d) Poor (e) Terrible

**26. Did you take naps before Ramadan?**

(a) Yes-usually (b) Yes-sometimes (c) No-never

**27.** **Did you take naps during ramadan?**

(a) Yes-usually (b) Yes-sometimes (c) No-never

**28.** **Do you take naps after ramadan?**

(a) Yes-usually (b) Yes-sometimes (c) No-never

1. **How much sleep did you get every night, on average before Ramadan?**

(a) Less than 4 hours (b) 4-6 hours

(c) 6-8 hours (d) More than 8 hours

1. **How much sleep did you get every night, on average during Ramadan?**

(a) Less than 4 hours (b) 4-6 hours

(c) 6-8 hours (d) More than 8 hours

1. **How much sleep do you get every night, on average after Ramadan?**

(a) Less than 4 hours (b) 4-6 hours

(c) 6-8 hours (d) More than 8 hours

# Did you have trouble falling asleep before Ramadan?

(a) Yes-always (b) Yes-sometimes

(c) Yes-Usually (d) No-never

# Did you have trouble falling asleep during Ramadan?

(a) Yes-always (b) Yes-sometimes

(c) Yes-Usually (d) No-never

# Do you have trouble falling asleep after Ramadan?

(a) Yes-always (b) Yes-sometimes

(c) Yes-Usually (d) No-never

# Do you have trouble getting back to sleep once you're awake?

(a) Yes-always (b) Yes-sometimes

(c) Yes-usually (d) No-never

# How anxious did you feel before Ramadan?

(a) Extremely (b) Moderately

(c) Slightly (d) Not at all

# How anxious did you feel during Ramadan?

(a) Extremely (b) Moderately

(c) Slightly (d) Not at all

# How anxious have you felt after Ramadan?

(a) Extremely (b) Moderately

(c) Slightly (d) Not at all

# How happy did you feel in before Ramadan?

(a) Extremely (b) Moderately

(c) Slightly (d) Not at all

# How happy did you feel during Ramadan?

(a) Extremely (b) Moderately

(c) Slightly (d) Not at all

# How happy have you felt after Ramadan?

(a) Extremely (b) Moderately

(c) Slightly (d) Not at all

# How stressed did you feel before Ramadan?

(a) Extremely (b) Moderately

(c) Slightly (d) Not at all

# How stressed did you feel during Ramadan?

(a) Extremely (b) Moderately

(c) Slightly (d) Not at all

# How stressed have you felt after Ramadan?

(a) Extremely (b) Moderately

(c) Slightly (d) Not at all

# How healthy did you feel before Ramadan?

(a) Extremely (b) Moderately

(c) Slightly (d) Not at all

# How healthy did you feel during Ramadan?

(a) Extremely (b) Moderately

(c) Slightly (d) Not at all

# How healthy did you feel after Ramadan?

(a) Extremely (b) Moderately

(c) Slightly (d) Not at all

# How tired did you feel before Ramadan?

(a) Extremely (b) Moderately

(c) Slightly (d) Not at all

# How tired did you feel during Ramadan?

(a) Extremely (b) Moderately

(c) Slightly (d) Not at all

# 50. How tired have you felt after Ramadan?

(a) Extremely (b) Moderately

(c) Slightly (d) Not at all

# 51. Did you have any headaches before Ramadan?

(a) Yes-regularly (b) Yes-sometimes (c) No

# 52. Did you have any headaches during Ramadan?

(a) Yes-regularly (b) Yes-sometimes (c) No

# 53. Do you have any headaches after Ramadan?

(a) Yes-regularly (b) Yes-sometimes (c) No

**54. How often do you brush your teeth?**

(a) Once a day (b) Twice a day (c) More than twice a day

(d) Occasionally (e) Never

**55. What do you use for cleaning your teeth/Mouth ?**

(a) Miswak (b) Powder (c) Toothpaste

(d) Gargling (e) Mouthwash (f) None of the above

**56. Have you been diagnosed with any of these sexually transmitted diseases?**

(a) Chlamydia (b) Gonorrhea (c) Genital herpes

(d) Genital warts (e) HIV (f) Syphilis

(g) None of the above

**57. How many times have you visited your doctor in the past year?**

(a) 0-3 (b) 3-6 (c) >6

**58. How many major surgeries (requiring anesthesia) have you had in your life?**

(a) 0-3 (b) 3-6

(c) 6-9 (d) >10

**59. Have you been vaccinated with following vaccines? If yes, then please mention the vaccination date in the text box if known.**

(a) BCG (b) MMR (c) Polio

(d) Hepatitis B (e) COVID-19 (f) Rabies

(g) Other (h) None of the above

**References**

Clarke, K., & Ainsworth, M. (1993). A method of linking multivariate community structure to environmental variables. *Marine Ecology-Progress Series*, *92*, 205-205.

Gu, Z., Eils, R., & Schlesner, M. (2016). Complex heatmaps reveal patterns and correlations in multidimensional genomic data. *Bioinformatics*, *32*(18), 2847-2849.

Oksanen, J., Blanchet, F. G., Kindt, R., Legendre, P., Minchin, P. R., O’hara, R.,…Wagner, H. (2013). Package ‘vegan’. *Community ecology package, version*, *2*(9), 1-295.

Taylor, M. (2014). sinkr: A Collection of Functions Featured on the Blog'me nugget'. *R package version 1.0*.

Wilke, C. O. (2021). ggridges: Ridgeline Plots in'ggplot2'. R package version 0.5. 3. In.
